# Integrated Performance Metrics of Porous Carbon Toward Practical Supercapacitor Devices

**DOI:** 10.1007/s40820-026-02069-z

**Published:** 2026-01-26

**Authors:** Yuting Song, Sicheng Fan, Zerui Yan, Dafu Tang, Xiang Gao, Jiawei Guo, Yunlong Zhao, Qiulong Wei

**Affiliations:** 1 State Key Laboratory of Physical Chemistry of Solid Surface, College of Materials, Fujian Key Laboratory of Surface and Interface Engineering for High Performance Materials, Xiamen University, Xiamen, 361005 People’s Republic of China; 2 Dyson School of Design Engineering, Imperial College London, London, SW72BX UK

**Keywords:** Supercapacitors, Energy density, Activated carbon, *E*-tool, New descriptor

## Abstract

The scientific communities in both academia and industry are devoted to increasing energy density of supercapacitor devices, including investigating the relationship between carbon structure and capacitance of various activated carbon (AC) materials. However, most reported capacitance values are measured solely at the material level, which are difficult to directly translate into achievable energy densities for practical supercapacitor devices. In this work, we assemble supercapacitor pouch cells to reveal the insight relationships between the capacitance and porosity of AC materials and the optimal amount of electrolyte at the device level. Concurrently, a guidance on the required amount of electrolyte is provided, indicating that both the specific capacitance and porosity of AC materials collectively determine the energy density of a practical device (*E*_device_). Furthermore, we develop a computational *E*-tool for directly predicting *E*_device_ at an early stage of material-level electrochemical testing. Finally, we propose a new descriptor (η) that incorporates both the capacitance and porosity parameters of AC materials, which displays a linear relationship with *E*_device_. This study provides a reliable *E*-tool and η for accelerating the development of advanced charge storage mechanisms and carbon materials for practical supercapacitor devices.

**Supplementary Information:**

The online version contains supplementary material available at 10.1007/s40820-026-02069-z.

## Highlights

- This work establishs a guidance of required amount of electrolyte for activated carbons in supercapacitor devices.
- A novel *E*-tool is provided for predicting the energy density of supercapacitor devices via the inputting of intrinsic parameters of activated carbons.
- A new descriptor η, that integrates capacitance and porosity of activated carbon electrode, is able to quickly evaluate the energy density of supercapacitor devices.

**Supplementary Information:**

The online version contains supplementary material available at 10.1007/s40820-026-02069-z.

## Graphical abstract

**Figure:**
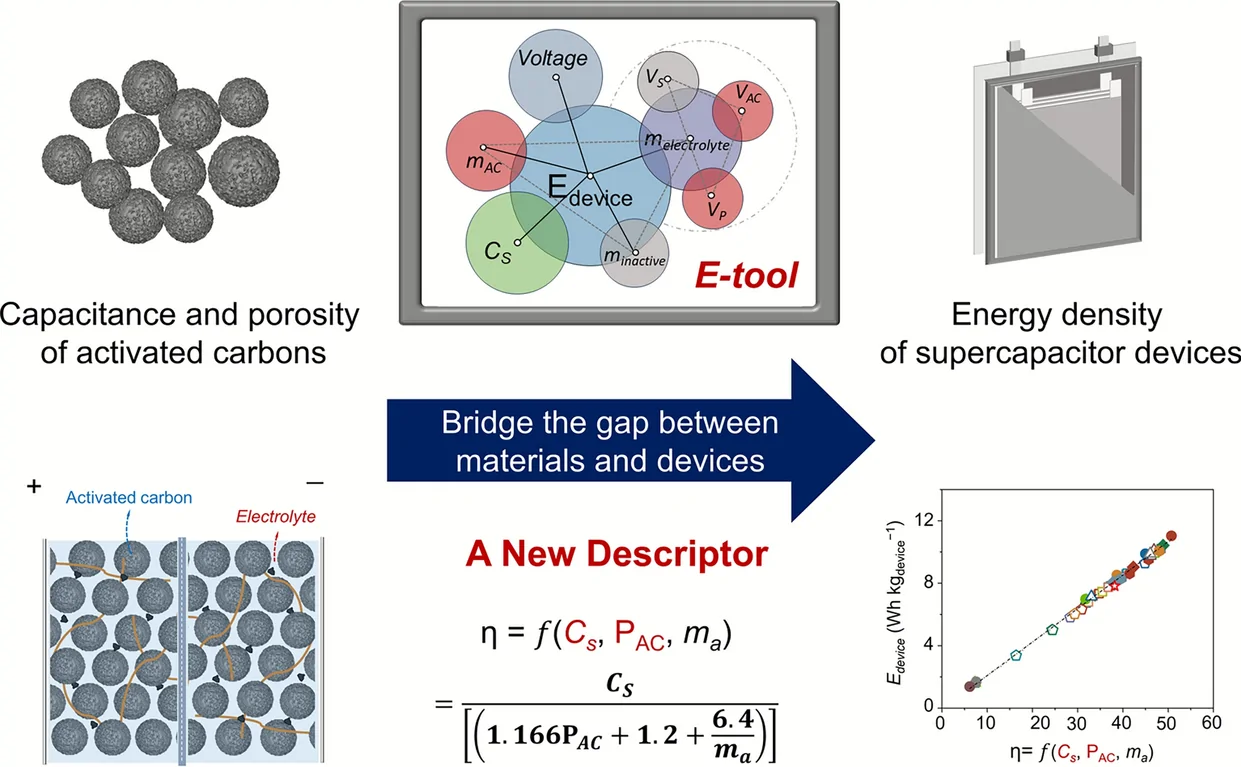

## Introduction

Electrochemical energy storage devices, including batteries and supercapacitors, are widely applied in mobile electronics, electric vehicles, and large-scale grids [1, 2]. Generally, battery and supercapacitor devices are composed of positive and negative electrodes (including active materials, carbon conductivities, binders, and current collectors), electrolytes, separators, and packages [3]. Extensive research efforts have been directed toward enhancing their energy densities, together with other properties such as power density, cycling life and safety [4–6]. Over the past three decades, lithium-ion batteries (LIBs) have achieved remarkable upgrades, accompanied by increased energy densities from 90 to 360 Wh kg_device_^−1^. The rapid development in energy density of device (*E*_device_, in Wh kg_device_^−1^) is not only attributed to the increased capacities of LiFe_0.5_Mn_0.5_PO_4_ [7], LiNi_x_Co_y_Mn_z_O_2_ [8], and Si/C materials [9, 10] compared with those of first-generation LiFePO_4_ [11], LiCoO_2_ [12], and graphite [5], but also the well-developed tools used to quickly calculate the energy density from the active materials to practical LIB devices (e.g., cylindrical, prismatic, and soft pouch cells) [13–15]. The tools were also developed into emerging lithium-metal batteries [16], which promote the realization of high *E*_device_ up to 503 and 547 Wh kg_device_^−1^ [17–19]. Emerging sodium-ion batteries also benefit from mature tools with well-designed roadmaps [20–23].

Compared with the rapid development of batteries, the *E*_device_ of supercapacitors is limited to 5–10 Wh kg_device_^−1^ [24]. Commercial supercapacitors use two pieces of activated carbon (AC) positive and negative electrodes, which are immersed in organic electrolytes [25, 26]. The AC stores charge through electric double layer (EDL) capacitive adsorption of ions in their nanopores [27]. The capacitance (*C*, in *F*) is used to describe the electrochemical performance of supercapacitor materials and devices. Thus, the E of supercapacitors largely depends on the capacitance (*C*) and operating voltage (*V*) [28], according to E = 0.5 × C × V^2^. Many efforts focused on enlarging the surface area (SA) of porous carbons to increase C according to C = (ε_r_ × ε_0_ × SA)/*d*, where *d* is the effective thickness of the double layers [29]. Unfortunately, the *E*_device_ of supercapacitors is still very limited even when the SA increases from 1500 to > 3000 m^2^ g^−1^ [30]. Recently, the relationships between C and carbon structures have become clear, that is the pore size and degree of disorder (small graphene-like domains) determine the capacitance of AC materials [31]. The established relationship between EDL charge storage and the carbon structure promotes the increase of *E*_device_ [27, 29, 32]. However, making practical devices from the laboratory material level to scale production is expensive and cumbersome, accompanying with considerable economic risk in industry. Therefore, many advanced carbon materials with increased EDL capacitive storage performance have been reported in the literature, but few of them are commercialized. To accelerate development from advanced mechanisms/theories to practical products, a straightforward tool for supercapacitors that bridges the gaps between active materials and device-level metrics is urgently needed but remains largely unexplored.

At present, many studies reported the energy densities of supercapacitors based on the mass of active materials [33, 34]. Nevertheless, the energy density based only on the active materials might lead to large overestimations of the electrochemical performance for practical supercapacitor devices. Gogotsi and Simon have noted the large energy gaps between material level and practical devices when low mass loadings of carbon materials were used [35]. However, compared with the mature tool for LIBs [13, 14, 36–38], an exact calculation methodology from the materials to supercapacitor devices has not yet been clearly provided. Unlike the “rocking chair” mechanism of LIBs in which Li^+^ ions are provided from cathode and intercalate into anode [39], the supercapacitor is based on the separation of cations and anions in the negative and positive electrodes [28]. Therefore, the charge carriers are supplied by the electrolyte of supercapacitors, which are highly related to the porosity and capacitance of AC materials. At the material level, the specific capacitance of AC material itself (*C*_*S*_) was easily measured and reported [31, 40]. However, when AC materials are applied to practical supercapacitor devices, a comprehensive descriptor that combines *C*_*S*_ with the required mass of electrolyte and other inactive parts has not yet been established.

To promote the development of supercapacitor devices, a major aim is to predict the *E*_device_ values at the material level. Herein, we establish an insight into the relationship between the accumulative pore volume of AC materials (P_AC_) and the required volume of electrolyte on the basis of practical supercapacitor pouch cells. We find that both the *C*_*S*_ of AC materials and the total volume of electrolyte required to fill the nanopores affect the electrochemical performance of supercapacitor devices. Subsequently, an *E*-tool (Supplementary file) is developed and provided, which is able to easily predict the *E*_device_ based on 43 AC materials from the reported literature. A new descriptor (η) that integrates the *C*_*S*_ and porosity of AC materials is used to quickly evaluate the electrochemical performance at the device level. This work provides clear guidelines for AC materials, emphasizing the balance of *C*_*S*_ and P_AC_, for increasing energy density of supercapacitors.

## Experimental Section

### Materials

Commercial AC materials were used for the preparation of double-side coated electrodes with a mass loading of 13.0 mg cm^−2^. The electrode composition consisted of 95 wt% AC, 3 wt% conductive carbon additive, and 2 wt% polyvinylidene fluoride (PVDF) binder, which were coated onto aluminum foil.

### Materials Characterization

The Brunauer‒Emmett–Teller (BET) specific surface area was measured via nitrogen (N_2_) adsorption‒desorption isotherms at 77 K after degassing under vacuum at 350 °C for 6 h. The morphology and microstructure were examined by scanning electron microscopy (SEM, SU-70). Cross-sectional SEM samples were prepared via a Leica EM TIC 3X ion beam cutter. True density data of AC were recorded on an AccuPyc II 1340 analyzer using Helium as analysis gas.

### Assembly of Supercapacitor Pouch Cells

For the assembly of supercapacitor pouch cells, rectangular electrodes (4.3 cm × 5.6 cm) were stacked in an 11-negative/10-positive configuration separated by separators (NKK TF4030 cellulose separators). The assembly process was completed in an argon-filled glove box (H_2_O < 0.1 ppm, O_2_ < 0.1 ppm). The supercapacitor pouch cells were assembled using the electrolyte of 1 M Net_4_BF_4_ in ACN with different volumes or different concentrations (0.25, 0.5, 1.0, and 1.5 M). The electrolyte was purchased from DoDoChem.

### Electrochemical Characterization

All the electrochemical tests were performed in a climate-controlled chamber at 25 °C. The rate capability and cycling performance were evaluated via a NEWARE CE-6008A-5V100A-H battery testing system. The coin cells and pouch cells were tested in the voltage window of 0–2.7 V.

## Results and Discussion

### Relationship between Porosity of AC and Volume of Electrolyte in Supercapacitor Devices

**Fig. 1:**
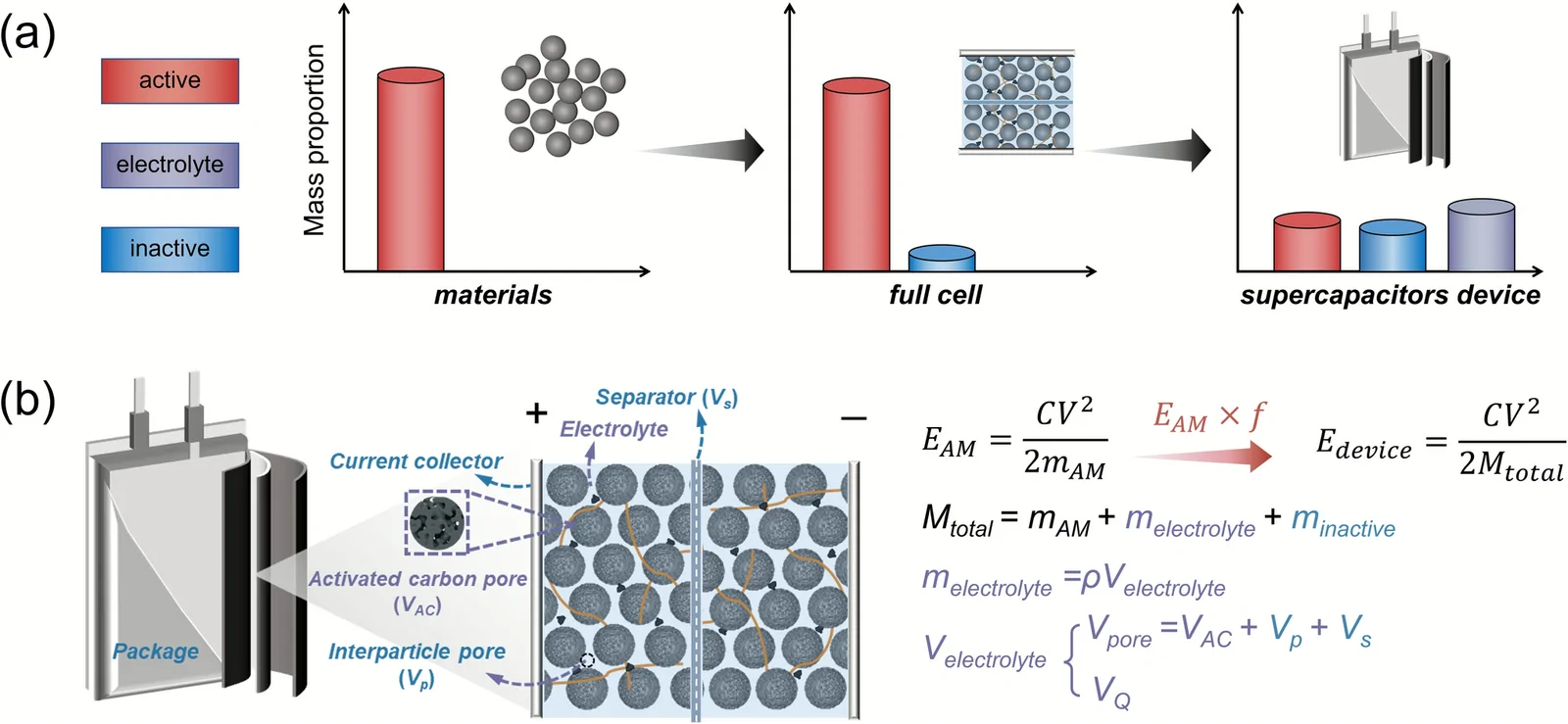
**a** Mass distributions of the considered components of supercapacitors at the material, full cell, and device levels. **b** Schematic of the inner structure of supercapacitor devices

The AC materials are made into electrodes by mixing conductive carbon additives and binders [34, 41]. Then, 2-electrode or 3-electrode cells are assembled to evaluate their electrochemical performance. At the material level (Fig. 1a), the capacity is obtained based on the weight of the active material itself. At the level of 2-electrode full cell, the capacity and energy density are calculated based on total masses of the positive and negative electrodes, which include the weights of the AC and inactive components of carbon additives and binders [33]. For industrial manufacturing, AC materials are produced in kilogram or ton quantities and made into double-side coated thick-film electrodes. Then, the supercapacitor devices are assembled by stacking the same AC films as the negative and positive electrodes, injecting the electrolyte and encapsulating into packages (*e.g.,* soft-package, prismatic or cylindrical configurations). Therefore, at the device level (Fig. 1a), all the masses of each component need to be considered, including the AC materials, carbon additives, binders, electrolyte and other inactive components of current collectors, separator, and package [35, 42, 43]. Thus, there is a large mass gap between material level and particle supercapacitor devices.

The conversion factor (*f*) for the energy density from the material level to practical supercapacitor devices (Fig. 1b) is calculated via Eqs. 1 − 4 [44, 45]:

1 $$ E_{AC} = \frac{1}{8} \times C_{S} \times V^{2} $$

2 $$ E_{device} = f \times E_{AC} $$

3 $$ f =\frac{{E_{AC} }}{{E_{device} }} =\frac{{m_{AC} }}{{M_{total} }} $$

4 $$ M_{total} = m_{AC} + m_{electrolyte} + m_{inactive} $$

*V* is the voltage window, and *M*_*total*_ is related to the total mass of the device, including $m_{{{\mathrm{AC}}}}$ (the mass of AC in the negative and positive electrodes), the mass of the injected electrolyte $m_{electrolyte} = V_{electrolyte} \times \rho_{electrolyte}$, and $m_{inactive}$ (including the package, current collectors, separator, binders, and carbon additives).

The amount of electrolyte required for a supercapacitor is highly related to the capacitance and porosity of the AC material. In a supercapacitor device (Fig. 1b), according to Eq. 5, the total pore volume (V_pore_) includes the pore volume of the AC material (V_AC_), the pores between stacked particles (${\mathrm{V}}_{{\mathrm{P}}}$) and the pores of the separator (V_S_). According to Eq. 6, the V_AC_ refers to the adsorption of electrolyte in the open nanopores of AC materials, while the P_AC_ is measured through isothermal N_2_ adsorption‒desorption measurements. The porosity of the electrode (P_electrode_) is obtained according to Eq. 7, where $\rho_{AC}$ is the true density of the AC materials. Thus, the V_P_ is calculated via Eq. 8. The Vs is calculated via Eq. 9, which depends on the properties of the separator, including its porosity (P_s_), thickness (*h*_s_) and total area used in devices (*A*_s_).

5 $$ {\mathrm{V}}_{{{\mathrm{pore}}}} \, = \,{\mathrm{V}}_{{{\mathrm{AC}}}} \, + \,{\mathrm{V}}_{{\mathrm{P}}} \, + \,{\mathrm{V}}_{{\mathrm{S}}} $$

6 $$ V_{AC} = P_{AC} \times m_{AC} $$

7 $$ P_{electrode} = 1 - \frac{{\rho_{electrode} }}{{\rho_{AC} }} $$

8 $$ V_{P} = V_{electrode} \times P_{electrode} - V_{AC} $$

9 $$ V_{S} \, = P_{s} \times h_{s} \times A_{s} $$

Additionally, the electrolyte in supercapacitor devices provides the cations and anions that are separately stored in two electrodes. Thus, the volume of electrolyte for charge storage (V_Q_) depends on the capacitance of the AC, according to Eq. 10,

10 $$ V_{Q} = \frac{C \times V}{{F \times \rho_{electrolyte} }} $$

where *C* is the capacitance of the device, *V* is the rated voltage, F is the Faraday constant (96,485 C mol^−1^), and ${\uprho }_{{{\mathrm{electrolyte}}}}$ is the density of the electrolyte (mol L^−1^).

To investigate the required V_electrolyte_, a supercapacitor pouch cell composed of 10 positive electrodes and 11 negative electrodes (Fig. S1) is designed and assembled. The P_AC_ was 0.65 mL g^−1^ according to isothermal N_2_ adsorption‒desorption measurements (Fig. S2 and Table S2). The ${\uprho }_{{{\mathrm{electrode}}}}$ is calculated based on the mass loading of 13.0 mg cm^−2^ and a thickness of 200 μm under a double-side coating (as measured by the cross-sectional SEM image in Fig. S3, except for the thickness of the Al foil). The true density of the AC (ρ_AC_) was measured through helium pycnometry (Fig. S4) [46, 47]. The information of the separator is listed in Table S1, where P_S_ is 73% and *h*_s_ is 30 μm. The electrolyte is 1 M Net_4_BF_4_ in ACN. Therefore, as described in Fig. S1, the specific values of V_AC_ = 4.04 mL, V_S_ = 1.36 mL, V_P_ = 2.66 mL, V_pore_ = 8.10 mL and V_Q_ = 3.80 mL are calculated on the basis of the AC electrodes.

**Fig. 2:**
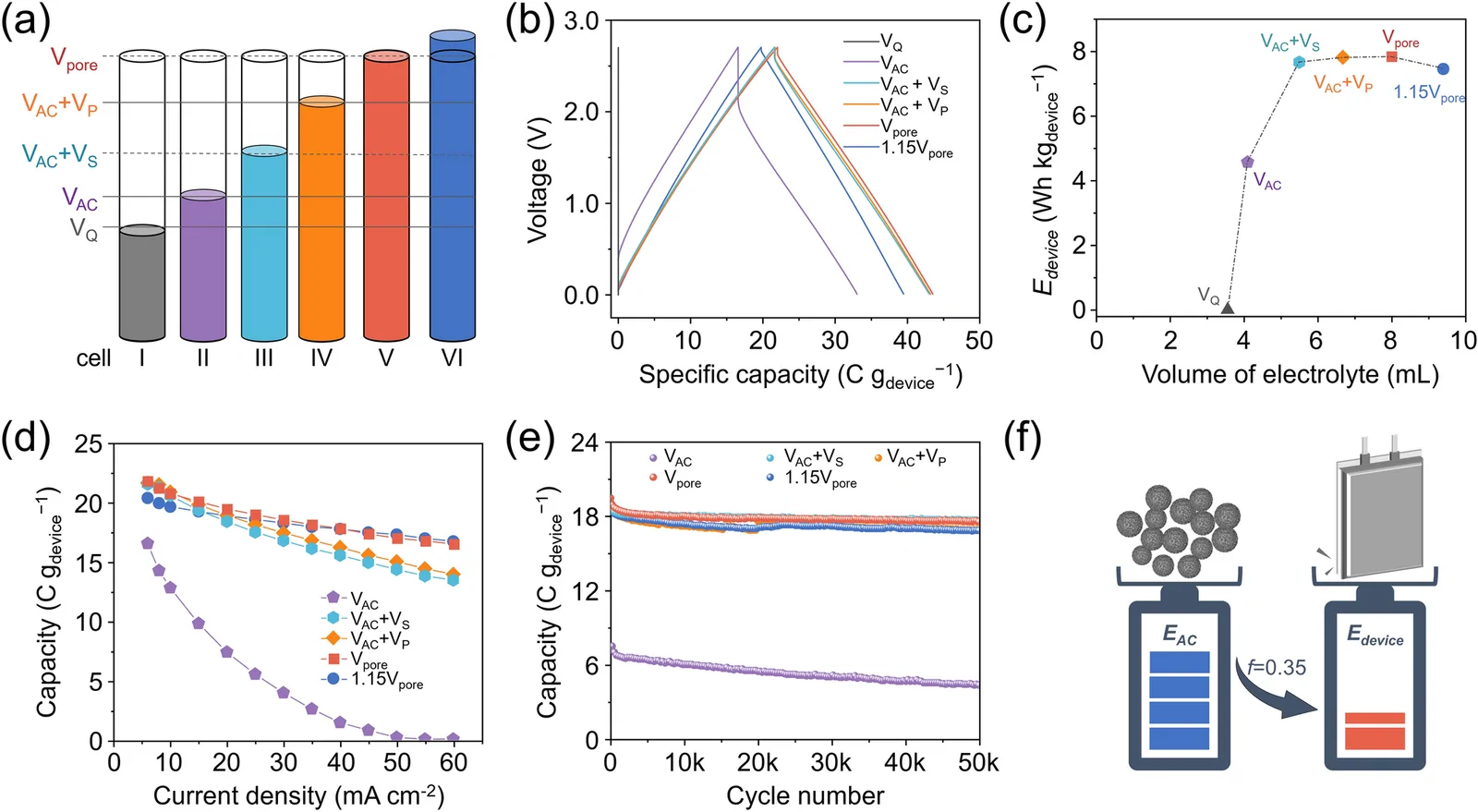
**a** Relation between the pore volume and the electrolyte volume in supercapacitor devices. **b** Galvanostatic charge and discharge curves of supercapacitors at 6 mA cm^−2^. **c** Measured *E*_*device*_, **d** rate capabilities and **e** cycling performance of supercapacitors with different volumes of electrolyte. **f** Conversion factor of the as-assembled supercapacitor pouch cell, when V_electrolyte_ = V_pore_

Figure 2a shows the injections of different V_electrolyte_ for each cell, including V_Q_, V_AC_, V_AC_ + V_S_, V_AC_ + V_P_, V_pore_, and extra amounts (1.15V_pore_). The supercapacitor pouch cells were measured by galvanostatic charging and discharging (GCD) at 6 mA cm^−2^ (Figs. 2b and S5). The cell I is unable to operate when the V_electrolyte_ = V_Q_, showing no capacity. When V_electrolyte_ = V_AC_, the cell II shows linear GCD curves from the EDL capacitive charge storage. However, the GCD curves do not show the ideal triangular shape with a limited operating voltage because the amount of V_AC_ is not enough to completely wet the electrodes and separators [48]. Such poor wetting and insufficient ion availability lead to limited capacity and high resistances [49, 50]. When electrolyte volume is continuously increased, GCD curves of cells III, IV and V show the ideal triangle shapes, whereas cell V (V_electrolyte_ = V_pore_) has the highest capacity. However, when extra electrolyte is added (V_electrolyte_ = 1.15 V_pore_), the capacity decreases owing to the increased electrolyte mass for the whole devices.

The energy densities of the six cells are further calculated based on the total weight of the device (Fig. 2c). This result clearly shows that the energy density of the device depends on the amount of added electrolyte. The electrolyte should be enough to fully fill the pores (V_electrolyte_ = V_pore_), which enables the high *E*_device_. Rate performance of the supercapacitors is highly important and further compared (Figs. 2d and S6). Even through cells III, IV, and V have similar capacities at low current densities, their capacities at high currents are very different, indicating that the amount of electrolyte still influences the rate capacities of supercapacitor devices. The cells V and VI exhibit superior capacity retention at high current densities of 60 mA cm⁻^2^, indicating that a sufficient electrolyte quantity facilitates rapid charge/discharge kinetics in the device by enhancing ion transport. When V_electrolyte_ = V_pore_, cell V displays not only the highest *E*_device_ but also the best rate capabilities among the six pouch cells. Additionally, cells III to VI (Fig. 2e) have 50,000 stable cycles with negligible capacity losses, indicating that a sufficient amount of electrolyte does not affect the cycling stability. The above results reveal the critical role of the amount of electrolyte in determining the balance among the capacity, *E*_device_ and rate capabilities of particle supercapacitor devices.

Figure 2f shows the energy density based on the electrochemical testing of the coin cell (Fig. S7) and pouch cell (Fig. 2c). The weight proportions of the AC, electrolyte and inactive parts are 35%, 38%, and 27%, respectively, for pouch cell V. The *E*_AC_ calculated on coin cell and pouch cell is close to 21.80 Wh kg_AC_^−1^, and the *E*_device_ of cell V is 7.80 Wh kg_device_^−1^. Thus, the conversion factor of supercapacitors is approximately 0.35, which is much lower than that of 0.6 − 0.7 for LIBs [4, 13]. The *E*_device_ of our pouch cell is higher than that of commercial 100 F cylindrical supercapacitors (4.73 Wh kg_device_^−1^), primarily due to the lower packaging mass of the aluminum‒plastic film compared with that of metal casings (Fig. S8).

### Influence of Electrolyte Concentration on Supercapacitor Performances

**Fig. 3:**
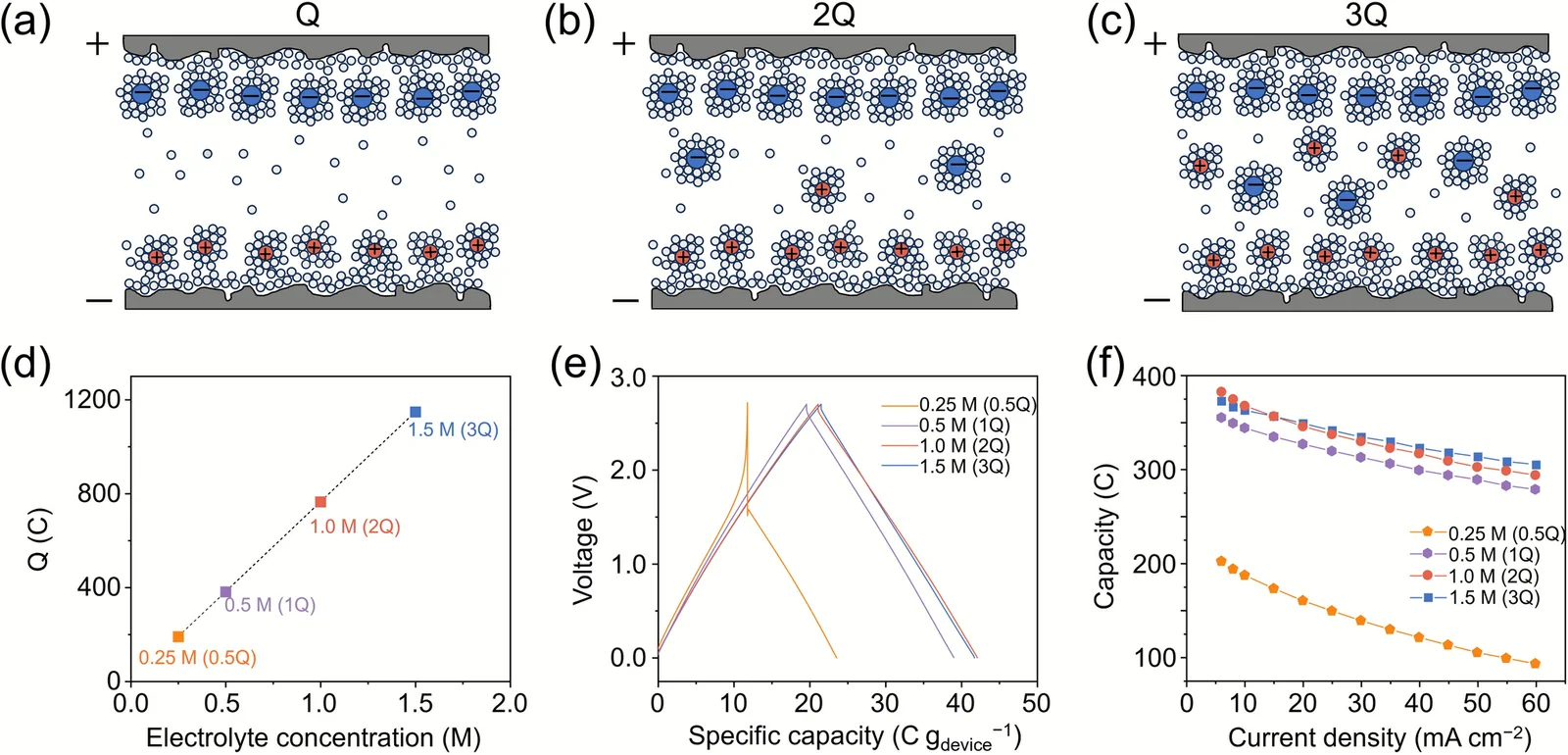
Schematic of the ion distributions for the EDL capacitive charge: **a** Q_electrolyte_ = Q, **b** Q_electrolyte_ = 2Q, **c** Q_electrolyte_ = 3Q. **d** Corresponding electric quantities for different concentrations of electrolytes. **e** Galvanostatic charge and discharge curves and **f** rate capabilities of supercapacitors with different concentrations of electrolytes

The electrolyte in supercapacitor provides the charge carriers between the negative and positive electrodes. In a charging process, the accumulation of electronic charge on the electrode surface is counterbalanced by an equivalent but oppositely charged double layer at the electrode‒electrolyte interface [51, 52]. Consequently, the ions in electrolyte need to meet the minimum charge compensation to ensure charge storage. However, in this situation, the concentration and ionic conductivity of bulk electrolyte are largely reduced, leading to insufficient charge storage (Fig. 3a) [44]. Thus, the extra number of ions are required in electrolyte to ensure high ionic conductivity (Fig. 3b, c). This implies that the minimal electrolyte volume required for charge compensation represents the lowest threshold for maintaining the operation of device. As discussed in Fig. 2, when 1 M Net_4_BF_4_ in ACN is used as electrolyte, the electrolyte volume sufficient for charge compensation (${\mathrm{V}}_{{\mathrm{Q}}}$) does not fully occupy the available pore volume of the electrode. As a result, a fraction of the active sites is not wetted due to insufficient solvent. In this part, electrolytes with various concentrations of 0.25, 0.5, 1.0, and 1.5 M are investigated (Table S3). The V_electrolyte_ was equal to V_pore_ (7.9 ± 0.1 mL). According to Eq. 10, the charge capacity for different concentrations of electrolyte refers to 0.5Q, 1Q, 2Q, and 3Q (Fig. 3d).

When V_electrolyte_ = V_pore_ for the 0.25 M electrolyte that less than the theoretical charge quantity (Q) (Figs. 3e and S9), its operating voltage is limited to ~ 1.5 V with very low capacity owing to the limited number of ions provided by the electrolyte for EDL capacitive charge storage of ions in nanopores. When the concentration increases to 0.5 M (Q), the pouch cell can operate at 2.7 V with increased capacity. An optimal electrochemical performance is achieved for the 1.0 M electrolyte (2Q), which displays the maximum utilization of AC and the electrolyte for EDL storage. Upon increasing the concentration to 1.5 M (3Q), no additional improvement is observed, as evidenced by the nearly overlapping GCD curves with those of cycling in 1.0 M electrolyte. Figure 3f shows the excellent rate capabilities up to a high current density of 60 mA cm^−2^ when 1 and 1.5 M electrolytes are used. The superior rate performance observed for concentrated electrolytes is directly attributed to their higher ionic conductivity (Fig. S10, Table S3). To ensure optimal capacitor operation, the 1.0 M electrolyte system (2Q) was identified as the most effective configuration, enabling the device to achieve both superior rate capability (Fig. S11a) and high energy density (Fig. S11b). Owing to the EDL charge storage mechanism, all the supercapacitor devices demonstrated excellent cycling stability (Fig. S11c). The above results (Figs. 2 and 3) provide guidance concerning the amount of electrolyte required to completely fill the nanopores of AC materials and maintain sufficient charge carrier availability to maximize the capacity and energy density of practical supercapacitor devices.

### Evaluating the Energy Density from AC Materials to Supercapacitor Devices

**Fig. 4:**
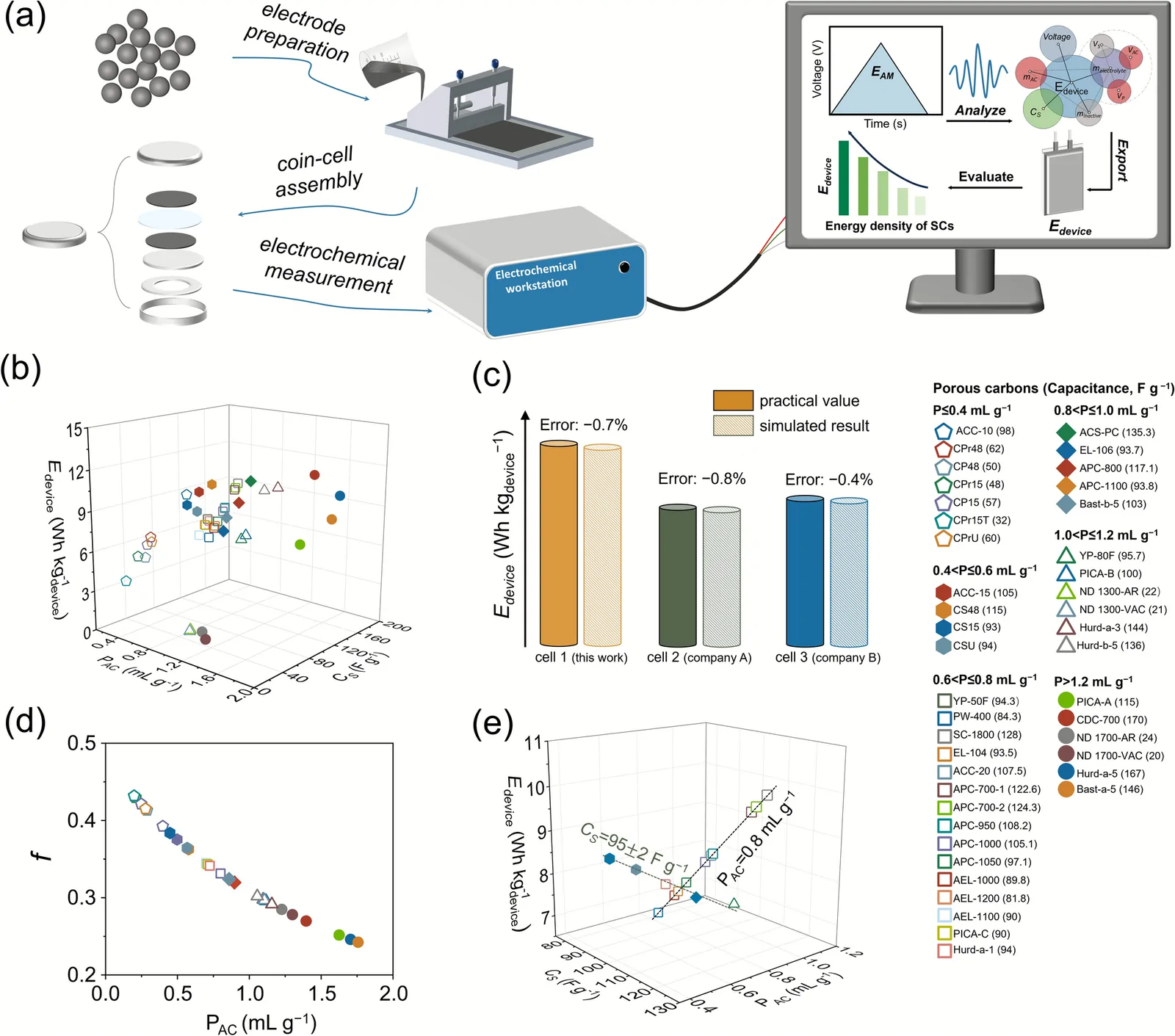
**a** Flowchart of *E*_device_ prediction from material to device levels. **b** Predictions of the *E*_device_ of 43 AC samples with different ${\mathrm{C}}_{{\mathrm{S}}}$ and P_AC_ from literatures (Table S4). **c** Validation of the *E*-tool based on practical supercapacitor devices. **d** Plots of the simulated conversion factor (*f*) *vs.* P_AC_. **e** 3D plot of the simulated *E*_device_* vs. C*_*S*_ and P_AC_

Our above experimental results reveal the relationships between the capacitance and porosity of AC materials and the optimal amount of electrolyte for determining the electrochemical performance of practical supercapacitor devices. Accordingly, an effective tool (*E*-tool for supercapacitor in Supporting Information) is provided to evaluate the energy density from AC materials to practical supercapacitor devices. On the basis of the electrochemical performance measured by coin cells (capacitance and operating voltage) and the physical properties of the AC material (P_AC_ and ρ_AC_), the *E*_device_ and *f* are able to be automatically output by this *E*-tool (Fig. 4a). Therefore, this E-tool helps establish a more comprehensive evaluation of AC materials for supercapacitor applications rather than only considering that their capacitances remain at the material level.

Recently, the electrochemical performance and porosity of various AC materials have been systematically reported for cycling in organic electrolytes. These data were adopted to discuss not only the influence of *C*_S_ but also the P_AC_ of AC materials on the overall *E*_device_. The V_electrolyte_ values related to the P_AC_ of AC materials are provided in the literature (Table S4). The 43 AC materials were divided into 6 groups according to their porosity. The true density of AC materials is usually in the range of 1.8–2.1 g cm⁻^3^ with a slight influence on the *E*_device_ (Fig. S12). Therefore, in the subsequent analyses, a true density of 2.0 g cm⁻^3^ was applied. The detailed parameters of the predicted supercapacitor cells are provided in Supporting Information.

The data were analyzed via our *E*-tool, generating a 3D plot (Fig. 4b) that illustrates the relationships among *E*_device_, *C*_*S*_, and P_AC_. Three supercapacitor devices, including two commercial cylindrical supercapacitors and our supercapacitor pouch cell, are analyzed to validate the accuracy of the *E*-tool (Fig. 4c). The detailed verification is described in Figs. S13 and S14. A comparison between the experimentally measured and predicted *E*_device_ values revealed excellent agreement, with less than 1% error. These results collectively demonstrate the reliability of the *E*-tool for evaluating supercapacitor performance.

The *f* strongly depends on the P_AC_ properties. A lower P_AC_ content reduces the electrolyte mass requirements, consequently increasing the *f* value. Among the 43 tested AC materials, the CPr15T [53] demonstrated the lowest pore volume (0.2 cm^3^ g^−1^), requiring minimal electrolyte volume in practical devices and resulting in the highest *f* value of 0.43 (Fig. 4d). The *E*_device_ distribution across 43 ACs shows no clear trend because of competing influences from the *C*_*S*_ and P_AC_. For a constant ${\mathrm{C}}_{{\mathrm{S}}}$ (95 ± 2 F g^−1^), *E*_device_ is inversely correlated with the cumulative pore volume (Fig. 4e). Reducing the accumulative pore volume from 1.10 to 0.45 cm^3^ g^−1^ increased *E*_device_ from 6.85 to 8.60 Wh kg_device_^−1^. Under the same pore volume of 0.80 cm^3^ g^−1^, the *E*_device_ linearly increases from 6.73 to 9.93 Wh kg_device_^−1^ with increasing capacitance of the AC materials.

### Descriptor for AC Materials for Evaluating the E_device_ of Supercapacitors

**Fig. 5:**
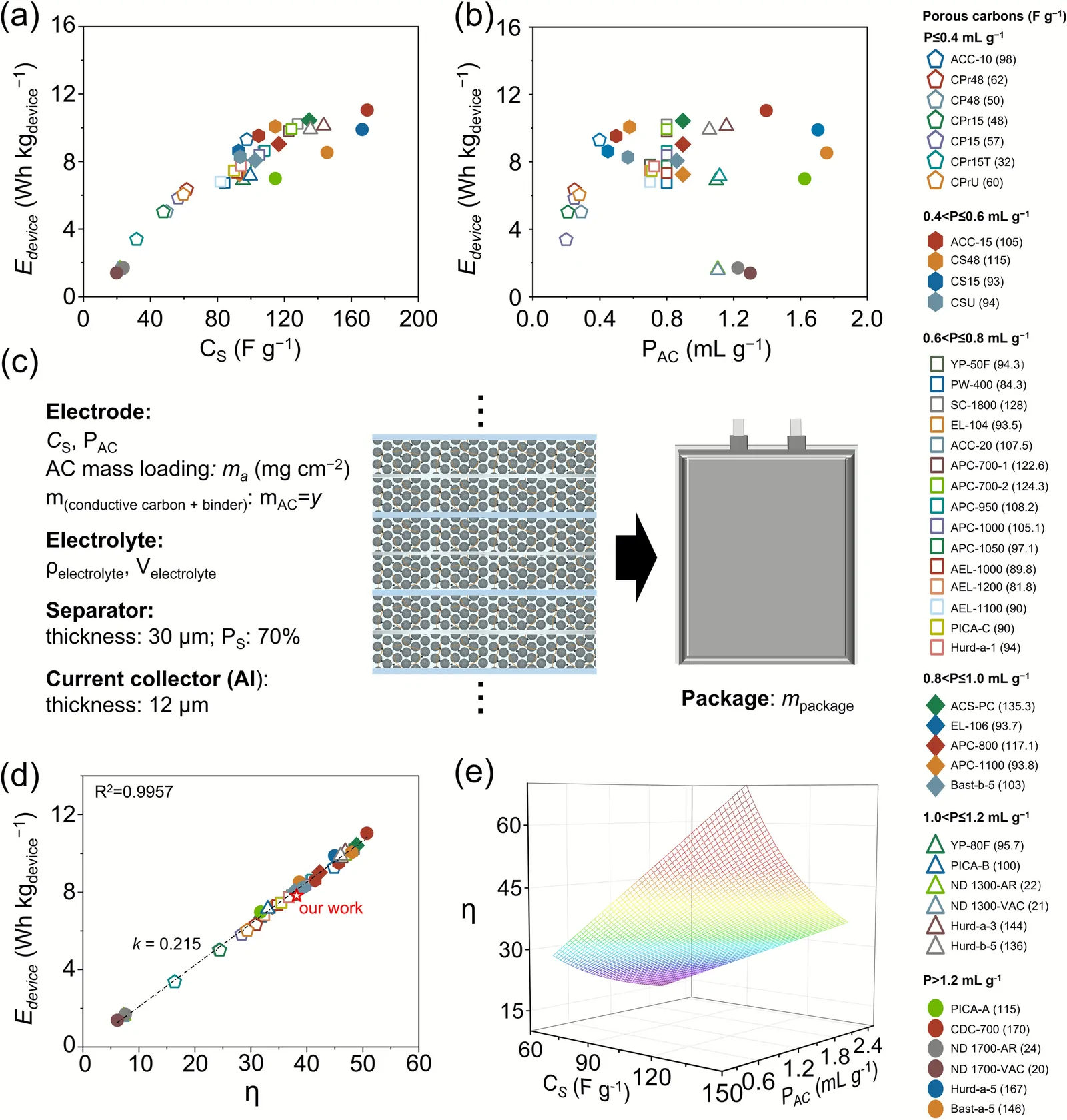
The plots of **a** *E*_device_ vs. *C*_*S*_ of AC materials and **b** *E*_device_ vs. P_AC_. **c** Schematic of supercapacitor devices, including the parameters of the electrode, electrolyte, separator, current collector and package. **d** Plot of *E*_*device*_* vs.* ${\upeta }$ when *m*_*a*_ = 13.0 mg cm^−2^. **e** 3D plots of η *vs. C*_*S*_ and P_AC_

There is not a clear correlation between the *E*_device_ and *C*_*S*_ (Fig. 5a),* E*_device_ and P_AC_ (Fig. 5b), and *E*_device_ and *SA* of AC materials (Fig. S15). To date, it does not have a descriptor for AC materials to determine the *E*_device_ from the material to device level. Based on above revealed relationship between P_AC_ and V_electrolyte_ (Figs. 2 and 3) and built-up *E*-tool (Fig. 4), we further propose a new descriptor (${\upeta }$) for AC materials, which is able to directly evaluate the final energy density when they are developed into assembled supercapacitor devices.

A detailed model of the supercapacitor cell is designed in Fig. 5c, where the *C*_S_, P_AC_, and mass loading of AC (*m*_a_) are comprehensively taken into Eq. 11 (the detailed calculation is provided in Supporting Information):

11 $$ E_{device} = f\left( {C_{S} ,{\mathrm{P}}_{AC} ,m_{a} } \right) = \frac{{C_{S} V^{2} }}{{8 \times 3.6 \times \left[ {\left( {1 + \frac{4.5}{{m_{a} }} + y} \right) + \rho_{e} \left( {1.35{\mathrm{P}}_{AC} + 0.175 + \frac{2.2}{{m_{a} }}} \right) + \zeta } \right]}} $$

*y* is the mass ratio of binder and conductive carbon, and $\rho_{e}$ is the density of electrolyte. Here, the ζ (m_package_/m_AC_) is a correction coefficient that is related to the type of device package. The influence of ζ on the *E*_device_ is illustrated in Fig. S16. The m_package_ of specific supercapacitor device is constant, which is not related to the properties of AC materials, while the influence of ζ would be decreased when larger supercapacitor cells are assembled.

Therefore, a new descriptor (${\upeta }$) is identified via Eq. 12, while the detailed calculation processes are provided in Supporting Information.

12 $$ {\upeta } = f\left( {C_{S} ,{\mathrm{P}}_{AC} ,m_{a} } \right) = \frac{{C_{S} }}{{\left[ {\left( {1 + \frac{4.5}{{m_{a} }} + y} \right) + \rho_{e} \left( {1.35{\mathrm{P}}_{AC} + 0.175 + \frac{2.2}{{m_{a} }}} \right)} \right]}} $$

When the AC/conductive carbon/binder ratio is 95:3:2 by weight, the *y* is calculated to be 0.053. The ρ_electrolyte_ of 1 M Net_4_BF_4_ in ACN is 0.8639 g mL^−1^, and the operating voltage is 2.7 V. Thus, the ${\upeta }$ is simplified to Eq. 13:

13 $$ {\upeta } = \frac{{C_{S} }}{{\left[ {\left( {1.166{\mathrm{P}}_{AC} + 1.2 + \frac{6.4}{{m_{a} }}} \right)} \right]}} $$

Figure 5d displays a linear relationship between *E*_device_ and ${\upeta }$ (when *m*_*a*_ = 13.0 mg cm^−2^), yielding a fitting slope of 0.215 and a coefficient of determination (R^2^) approaching 0.9957. Furthermore, the data based on our assembled supercapacitor pouch cells validate the linear relationship. Therefore, the new descriptor (${\upeta }$) is able to directly evaluate the properties of AC materials (*Cs*, P_AC_, and *m*_*a*_) for supercapacitor devices. A large value of ${\upeta }$ directly represents a high *E*_device_ when the AC materials are assembled into practical supercapacitor devices. Figure 5e shows the results of ${\upeta }$ *vs. C*s and P_AC_ (when *m*_*a*_ = 13.0 mg cm^−2^), while the high η values are achieved at high *C*_*S*_ combined with low P_AC_. According to Eq. 13, an increase in the mass loading of AC materials is also able to increase *E*_device_. The 3D plots illustrating the relationships among *m*_*a*_, *C*_*S*_ (or P_AC_), and η are provided in Fig. S17. However, when the *m*_*a*_ is higher than 28 mg cm^−2^, the increase in *E*_device_ is not significant (Fig. S18). Another important performance metric of a supercapacitor is fast charging and high-power delivery. Nevertheless, high mass loading means large electrode thickness, leading to increased transport distances of electron and ion and inefficient electrolyte penetration [50]. These factors collectively increase the overall polarization and decrease high-rate capabilities [54]. Consequently, it is important and full of challenge to increasing the electrode thickness and *E*_device_ without scarifying their capacitor-like fast kinetics [1, 55, 56].

## Conclusions

In summary, by revealing the electrochemical performance of supercapacitor pouch cells in terms of the relationship between the porosity of AC materials and the optimal volume of electrolyte, a guidance on the required amount of electrolyte is provided. The porosity of the AC materials is critical parameter for supercapacitor devices, while the electrolyte is necessary to fully fill the nanopores of AC materials. Furthermore, an *E*-tool is developed for predicting the *E*_device_ of practical supercapacitors on the basis of electrochemical testing of AC materials. The values of* E*_device_ are dependent not only on the *Cs* of the AC materials but also on their porosity (P_AC_). Therefore, the *C*_S_ measured via 2-electrode cell, and P_AC_ measured via isothermal N_2_ adsorption‒desorption, and the true density (or compaction density) of the AC materials is encouraged to provide. Lastly, we propose a new descriptor (η) that incorporates the *C*_*S*_ and P_AC_ of AC materials, which enables a linear relationship with *E*_device_. This work bridges the gap between the material level and the device level of supercapacitors. The *E*-tool and η offer valuable guidance for the design and synthesis of AC materials for increasing the energy density of practical supercapacitor devices. Moreover, we think this work provides a theoretical model for further AI-driven big data analysis of active materials for supercapacitors.

## Supplementary Information

Below is the link to the electronic supplementary material.

Supplementary file1 (DOCX 3972 KB)
