# Supplementary material for: Integrated Performance Metrics of Porous Carbon Toward Practical Supercapacitor Devices
Source: Nanomicro Lett. 2026 Jan 26;18:219. doi: 10.1007/s40820-026-02069-z (PMC12832599; doi:10.1007/s40820-026-02069-z)
Supplement: Supplementary file 1 — Supplementary file1 (DOCX 3972 KB) [file 40820_2026_2069_MOESM1_ESM.docx]

Supporting Information for

**Integrated Performance Metrics of Porous Carbon Toward Practical Supercapacitor Devices**

Yuting Song^1^, Sicheng Fan^1^, Zerui Yan^1^, Dafu Tang^1^, Xiang Gao^1^, Jiawei Guo^1^, Yunlong Zhao^2^, Qiulong Wei^1,^***

^1^State Key Laboratory of Physical Chemistry of Solid Surface, Fujian Key Laboratory of Surface and Interface Engineering for High Performance Materials, College of Materials, Xiamen University, Xiamen 361005, P. R. China

^2^Dyson School of Design Engineering, Imperial College London, London SW72BX, UK

*Corresponding author. E-mail: [qlwei@xmu.edu.cn](mailto:qlwei@xmu.edu.cn) (Qiulong Wei)

**S1 Calculation of the volume of electrolyte for supercapacitor pouch cells**

**S1.1 Volume of electrolyte based on the porosity of activated carbon (AC) electrodes**

The mass loading of the electrode was 13 mg cm^−2^ and a thickness of 200 μm (double sides, as measured by the cross-sectional SEM image in **Fig. S3**, except for the thickness of the Al foil). Thus, the ρ_electrode_ is$\text{ }$0.65 $\text{g }\text{cm}^{-\text{3}}$ (13 mg cm^−2^/200 μm).

The electrode area was 24 cm^2^ for each piece. The total volume of the electrodes: $\text{V}_{\text{electrode}}\text{=24×200×}\text{10}^{-\text{4}}\text{×}\text{ }\text{(10+11) =}\text{ }\text{10.08 }\text{mL}$.

The true density ($\text{ρ}_{\text{AC}}$) of the electrode powder was 1.94 g cm^−3^.

Porosity of the electrode: $\text{P}_{\text{electrode}}\text{ = (1}-\frac{\text{ρ}_{\text{electrode}}}{\text{ρ}_{\text{AC}}}\text{)×100\%}\text{ }\text{=}\text{ }\left（ \text{1}-\frac{\text{0.65}}{\text{1.94}} \right）\text{×100\%}\text{ }\text{=}\text{ }\text{66.5\%}$.

Hence, the pore volume of the electrode:

V_AC_+V_P_$\text{=}\text{ V}_{\text{electrode}}\text{×P}_{\text{electrode}}\text{ = 10.08×66.5\% = 6.7 }\text{mL}$.

The accumulative pore volume of the activated carbon is 0.65 mL g^−1^. The pore volume of AC material: V_AC_ = 0.65 mL g^−1^×(6.5 mg cm^−2^×24 cm^2^×2×21×95%)/1000 = 4.04 mL.

The stacking pore volume of the electrode is V_P_ = (V_AC_+V_P_) −V_AC_=6.7−4.04 = 2.66 mL.

The porosity of the stacking pore volume corresponds to V_P_/V_electrode_ = (2.66/10.08)×100 = 26.4%.

The separator also exhibits electrolyte absorption, which must be accounted for in total volume calculations. The total length of the separator is 103.2 cm. The volume of the separator is $\text{V}_{\text{Separator}}\text{ = 103.2}\text{×}\text{6}\text{×}\text{30}\text{×}\text{10}^{-\text{4}}\text{ = 1.86 mL}$, and the pore volume of the separator is

$\text{V}_{\text{S}}\text{ = }\text{V}_{\text{separator}}\text{×}\text{73\% = 1.8576}\text{×}\text{73\% = 1.36 }\text{mL}$.

$\text{V}_{\text{pore }}\text{ = }\text{V}_{\text{AC}}\text{+}\text{V}_{\text{P}}\text{+}\text{V}_{\text{S}}\text{ = 6.7+1.36 = 8.06 }\text{mL}$**.**

**S1.2 Volume of electrolyte is based on the Q of the device**

The voltage window of the supercapacitor is 0-2.7 V.

The rated capacitance of the supercapacitor is 137 F.

The Q of the device is $\text{Q}_{\text{theo}}\text{=}$137 F$\text{×}$2.7 V = 370 C, and related volume of electrolyte (1 M Net_4_BF_4_ in ACN) is [(370C)/(96485 C mol^−1^)$]\text{/}$(1 mol mL^−1^)$\approx$3.8 mL.

**V_Q_ = 3.8 mL.**

**S2 Prediction of *E_device_* based on 43 AC samples**

The mass loading of the AC electrode is 13 mg cm^-2^ (double sides, AC: conductive carbon: binder=95:3:2). Furthermore, the area of electrodes in soft-packaged pouch supercapacitors is 24 cm^2^. Furthermore, the electrolyte is 1 M Net_4_BF_4_ in ACN and separator is NKK TF4030 cellulose separators. The porosity of stacking pore in electrode is considered about 26%. The true density of different AC is assumed as 2 g cm^−3^.

The volume of AC (V_carbon_), V_carbon_ = m_AC_/$\text{ρ}$_ture_.

The pore volume of AC (V_AC_), V_AC_ = m_AC_*P_AC_

The volume of electrode, V_electrode_ = (V_AC_+V_carbon_)/ (1−26%).

The stacking pore volume (V_P_), V_P_ = V_electrode_*26%.

**S3 Calculation of descriptor (**$\text{η}$**)**

*E_device_*$=\frac{\frac{1}{8}C_{S}V^{2}*m_{AC}}{3.6M_{total}}$

$$M_{total}=m_{AC}+m_{electrolyte}+m_{inactive}$$

$$m_{incative}=m_{S}+m_{Al}+(m_{conductive crabon}+m_{binder})+m_{P}$$

The mass ratio between AC and other components can help simplify this equation. Because the current collector and separator exhibit approximately equal areas to the electrode, the mass ratio can simplify to the ratio of their respective area densities. The density of Al is well-established as ~2.7 g cm^−3^ with the Al foil thickness of 12 μm, the area density is calculated to be 3.24 mg cm^−2^. According to Table S1, the area density of separator is 1.263 mg cm^−2^. Assuming an AC mass loading of *m_a_* mg cm^−2^, the mass ratio between components becomes $m_{AC}$: $m_{Al}$: $m_{S}=$ m_a_: 3.24: 1.263. So, $m_{Al}=\frac{3.24}{m_{a}}*m_{AC}$, $m_{S}=\frac{1.263}{m_{a}}*m_{AC}$. Furthermore, the masses of conductive carbon and binder are determined by their respective ratios in the electrode slurry composition. We defined the electrode containing 95 wt.% AC powder, 3 wt.% conductive carbon, and 2 wt.% binder. Hence ($m_{conductive crabon}+m_{binder}$) =$\frac{\text{3\%+2\%}}{\text{95\%}}*m_{AC}$ = 0.053$m_{AC}$.

$$m_{electrolyte}=\rho_{e}V_{electrolyte}=\rho_{e}*\left( V_{AC}+V_{P}+V_{S} \right)=\rho_{e}*\left[ m_{AC}*P_{AC}+26\%*\left( \frac{\frac{m_{AC}}{\rho_{true}}+V_{AC}}{1-26\%} \right)+P_{S}*\frac{m_{S}}{\rho_{S}} \right]=\rho_{e}m_{AC}*(1.35P_{AC}+\frac{0.35}{\rho_{true}}+\frac{0.125P_{S}}{m_{a}})$$

$$M_{total}=m_{AC}+\left[ \rho_{e}m_{AC}*\left( 1.35P_{AC}+\frac{0.35}{\rho_{true}}+\frac{3P_{S}}{m_{a}} \right) \right]+\left[ \left( \frac{1.263}{m_{a}}*m_{AC} \right)+\left( \frac{3.24}{m_{a}}*m_{AC} \right)+\left( 0.053m_{AC} \right)+m_{P} \right]=m_{AC}\left[ 1.053+\rho_{e}*\left( 1.35P_{AC}+\frac{0.35}{\rho_{true}}+\frac{3P_{S}}{m_{a}} \right)+\frac{4.503}{m_{a}} \right]+m_{P}$$

Because the density of commercial electrolyte (1 M Net_4_BF_4_ in ACN) is 0.8639 g mL^−1^, $P_{S}=73\%$ (Table S1), and $\text{ρ}_{\text{true}}\text{≈}\text{2 g }$cm^−3^, so the equation can continue simplify to:

$M_{total}$=$m_{AC}\left( 1.166P_{AC}+\frac{6.4}{m_{a}}+1.2 \right)+m_{P}$

The E_device_:

$$E_{device}=\frac{\frac{1}{8}C_{S}V^{2}}{3.6*(1.166P_{AC}+\frac{6.4}{m_{a}}+1.2+\zeta)}$$

$$\eta=\frac{C_{S}}{(1.166P_{AC}+\frac{6.4}{m_{a}}+1.2)}$$

where $\zeta$ is the correction coefficient from the mass of package ($\zeta=\frac{m_{P}}{m_{AC}})$.

**S4 Supplementary Figures and Tables**


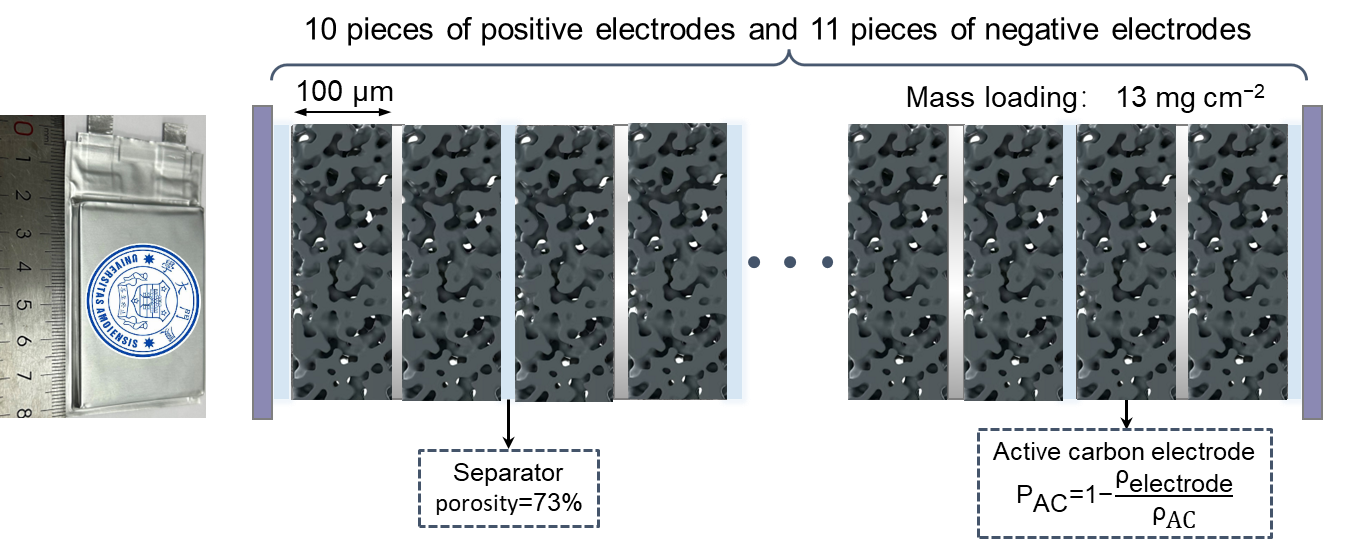


**Fig. S1** The design and assemble of supercapacitor pouch cell. This pouch cell is composed of 10 pieces of positive electrode and 11 pieces of negative electrode


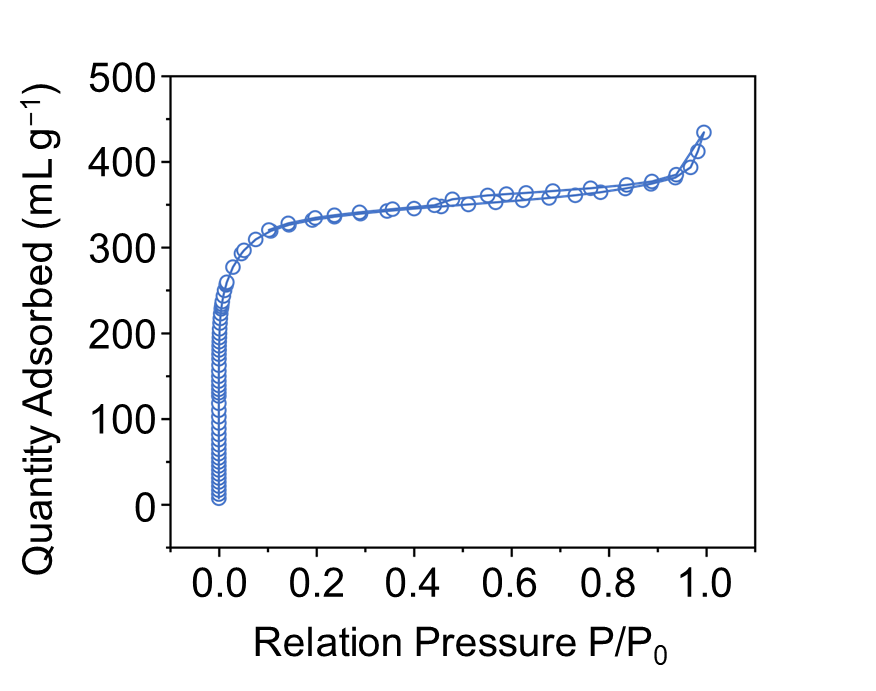


**Fig. S2** N_2_ adsorption‒desorption isotherms of the AC materials


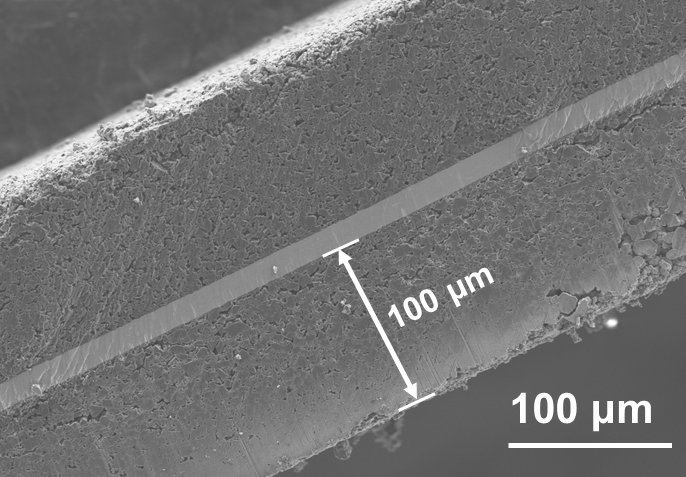


**Fig. S3** Cross‒sectional SEM images of the AC electrode (double-side coating)

**
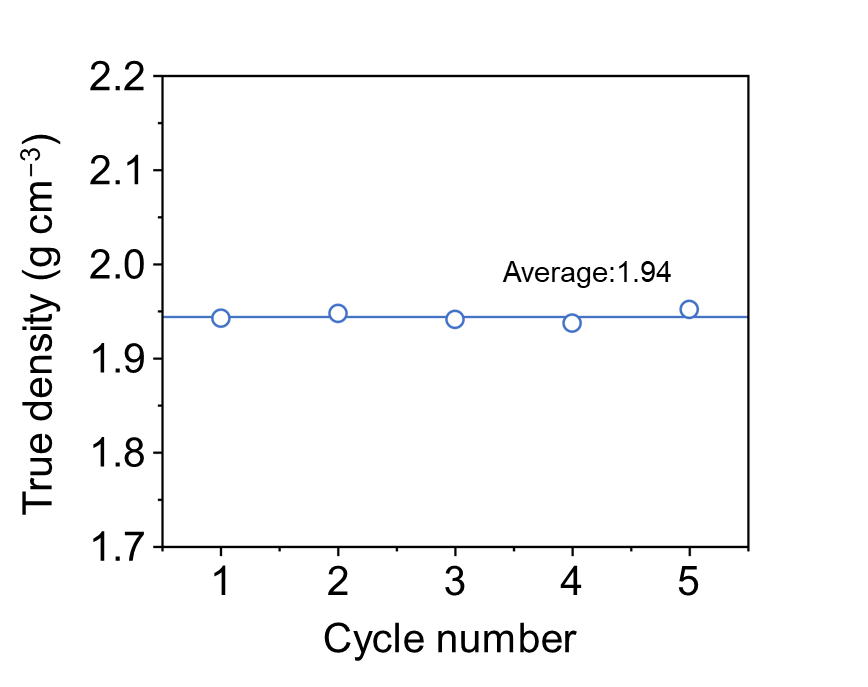
**

**Fig. S4** The true density of the AC materials

**
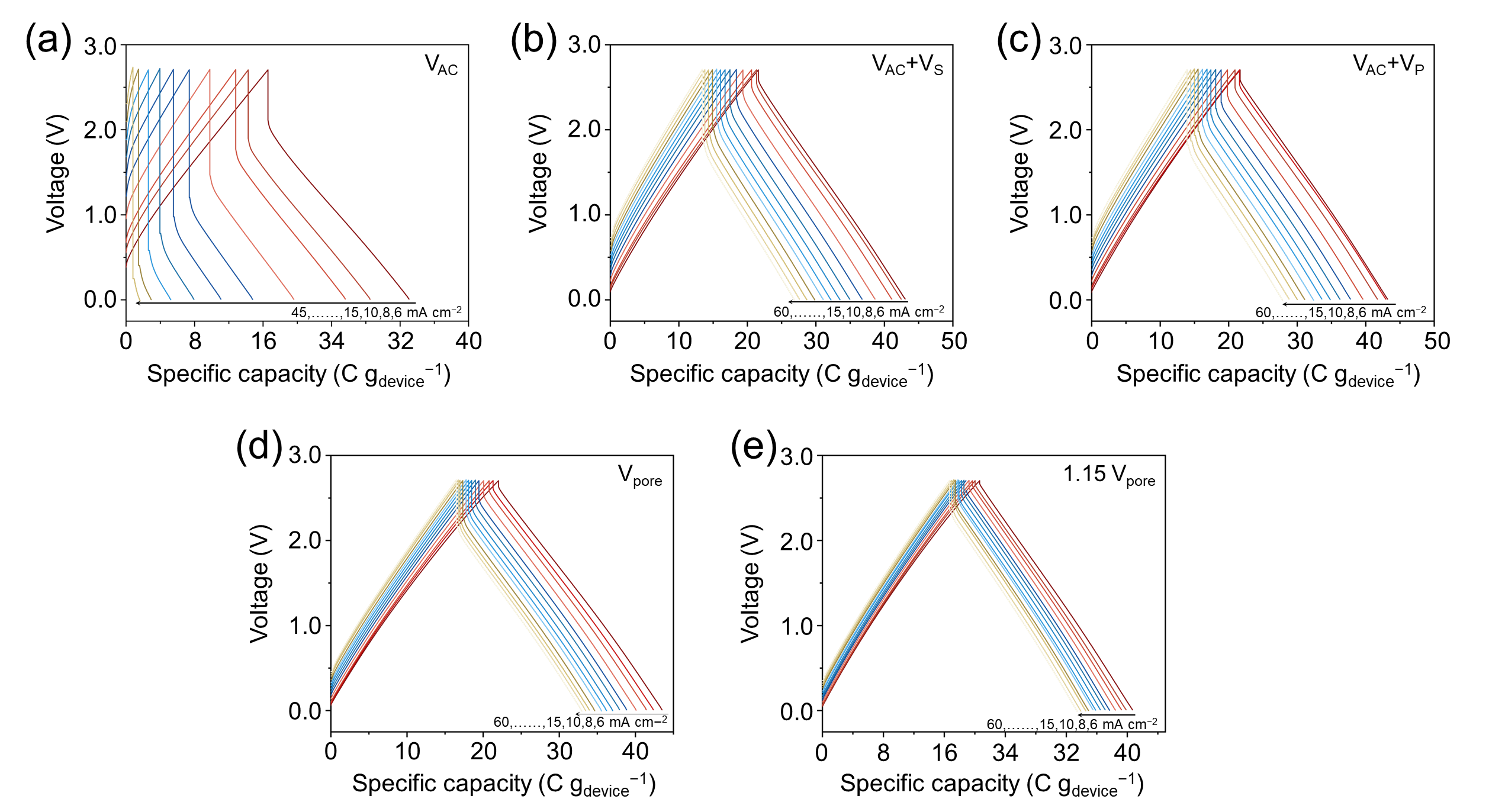
**

**Fig. S5** Charging and discharging curves at different current densities of supercapacitor pouch cell with volumes of electrolyte: **a** V_AC_, **b** V_AC_+V_S_, **c** V_AC_+V_P_, **d** V_pore_ and **e** 1.15 V_pore_

**
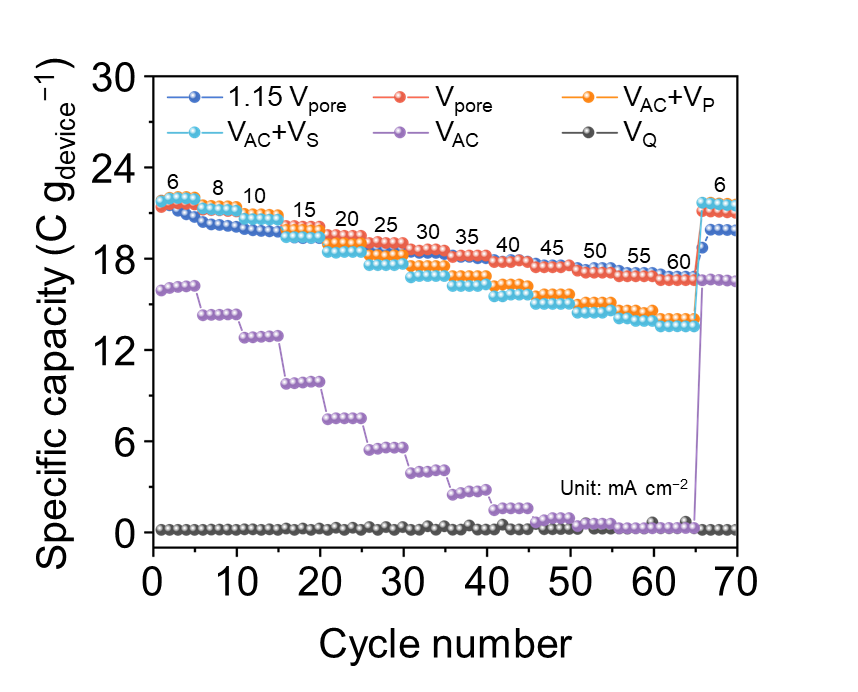
**

**Fig. S6** Rate performance of supercapacitor pouch cells with different volumes of electrolyte

**
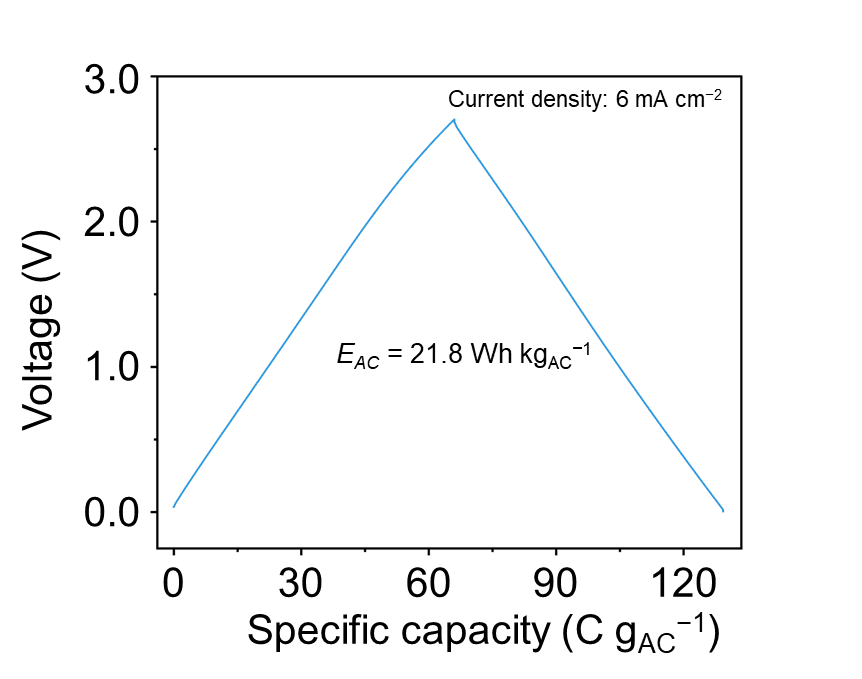
**

**Fig. S7** Charging and discharging curves of the supercapacitor measured by coin cell

**
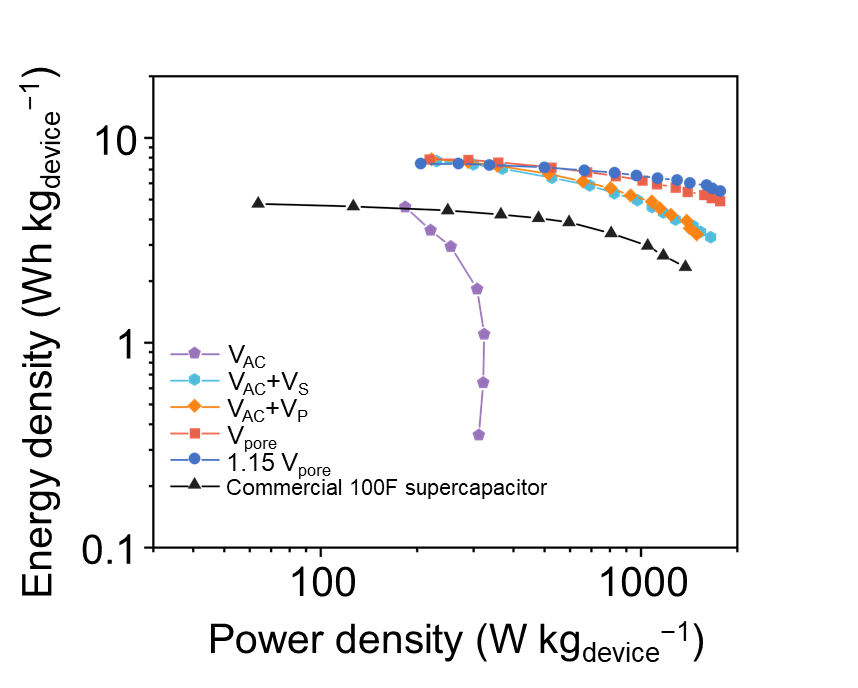
**

**Fig. S8** Ragon plots of as-assembled supercapacitor pouch cells with different volumes and commercial 100 F cylindrical supercapacitors

**
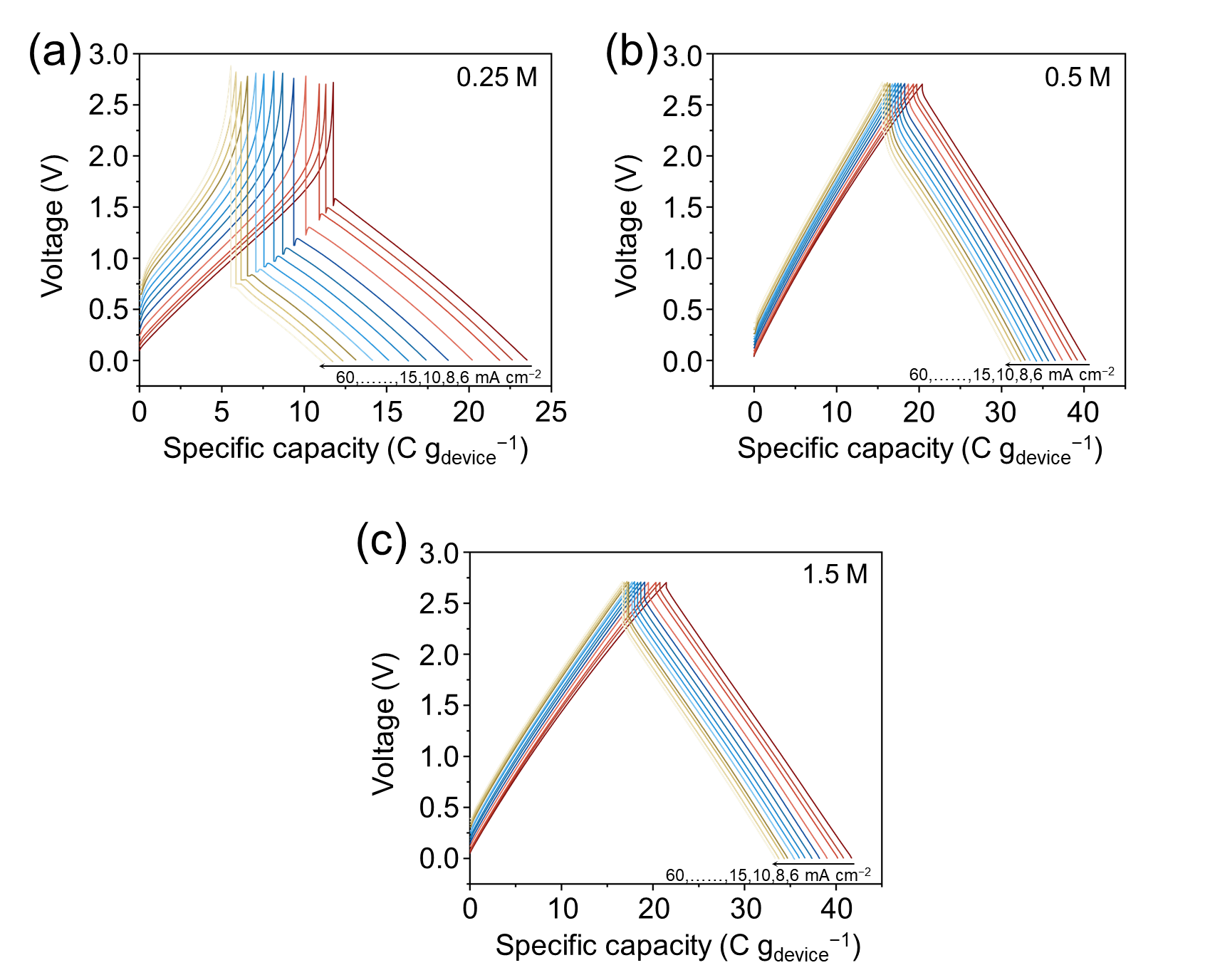
**

**Fig.** **S9** Charging and discharging curves of supercapacitor pouch cells with different electrolyte concentrations: **a** 0.25 M, **b** 0.5 M and **c** 1.5 M. The added electrolyte volume is consistent in 7.9$\pm$0.1 mL

**
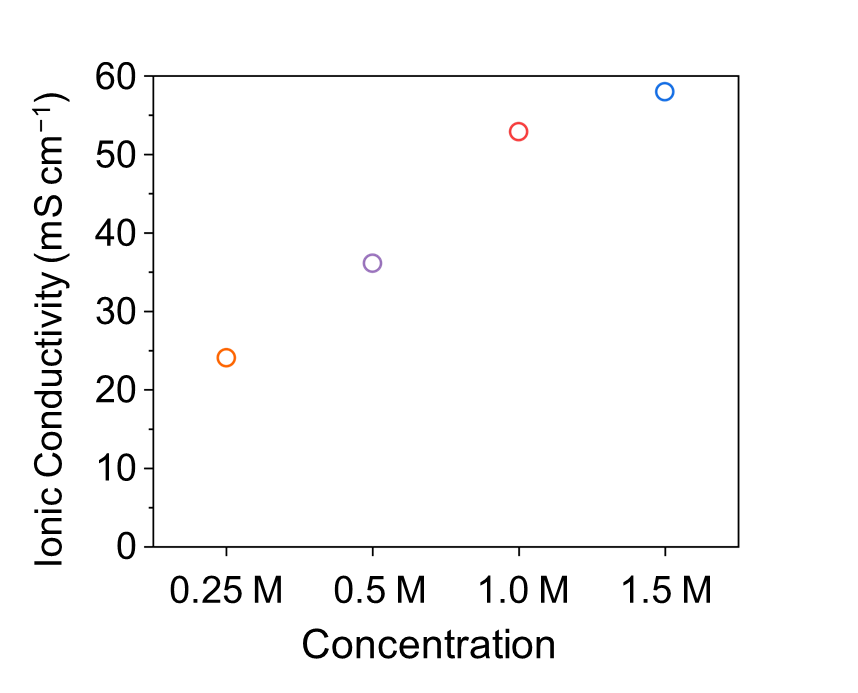
**

**Fig. S10** The ionic conductivities of electrolytes with different concentrations

The ionic conductivities of electrolytes with different concentrations were tested based the formula of $\sigma=L/RS$ [S1] at room tempreture, where $\sigma$ is ionic conductivity, R is the impedance, L is the distance between two parallel electrode and S is the area of the electrode. The L and S are constants for the same equipment. Additionally, the 0.01 M KCl ($\sigma_{KCL}=1.41$mS cm^−1^) was as nominal solution, and the ionic conductivities of electrolytes is calculated by following equation, $\sigma_{electrolyte}=\frac{R_{KCl}}{R_{electrolyte}}\times\sigma_{KCl}$. Thus, the ionic conductivities are calculated by the measured impendences of specific electrolyte.

**
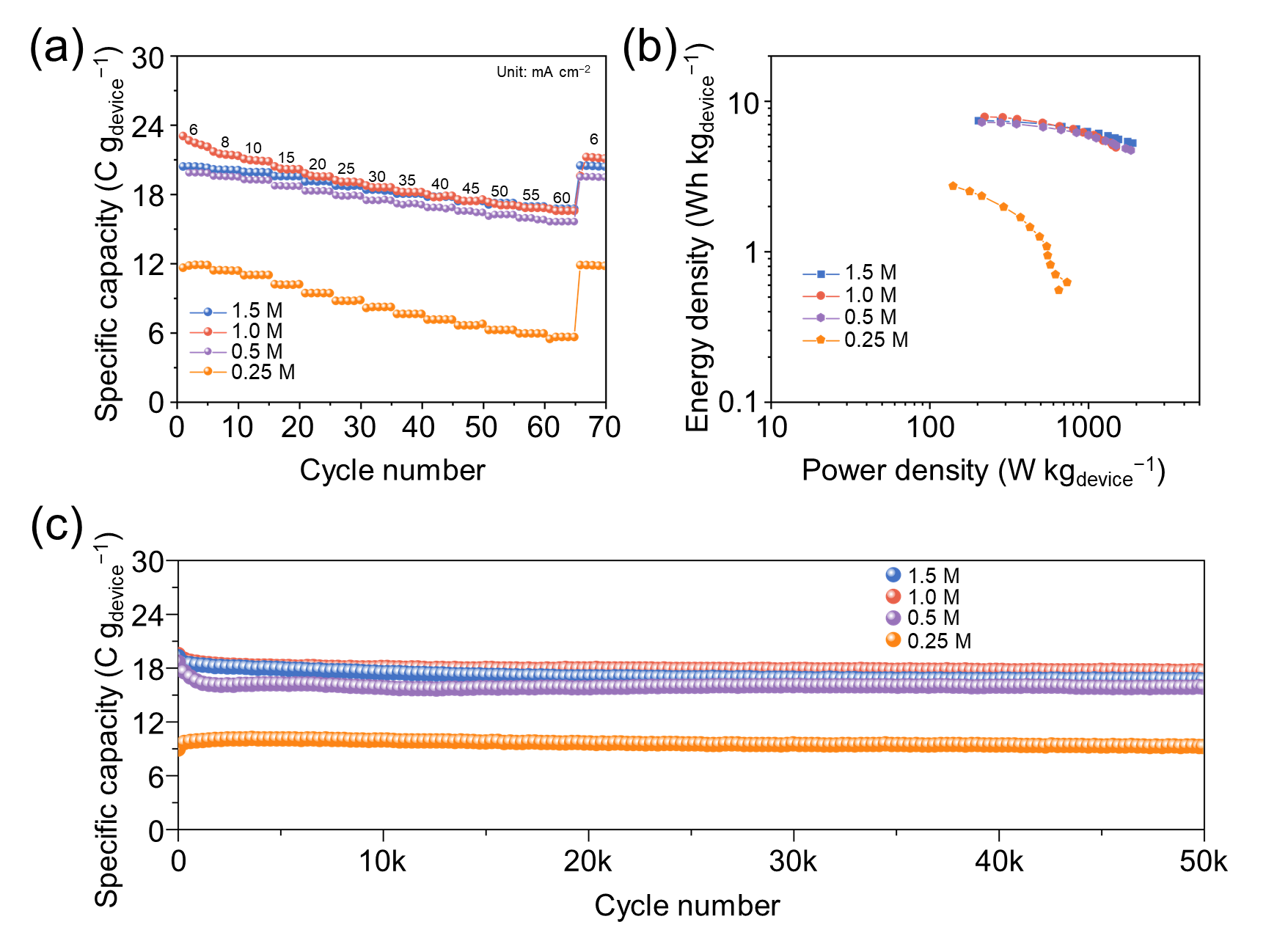
**

**Fig. S11** **a** Rate performance. **b** Ragone plots and **c** Cycling performance of supercapacitor pouch cells with different electrolyte concentrations


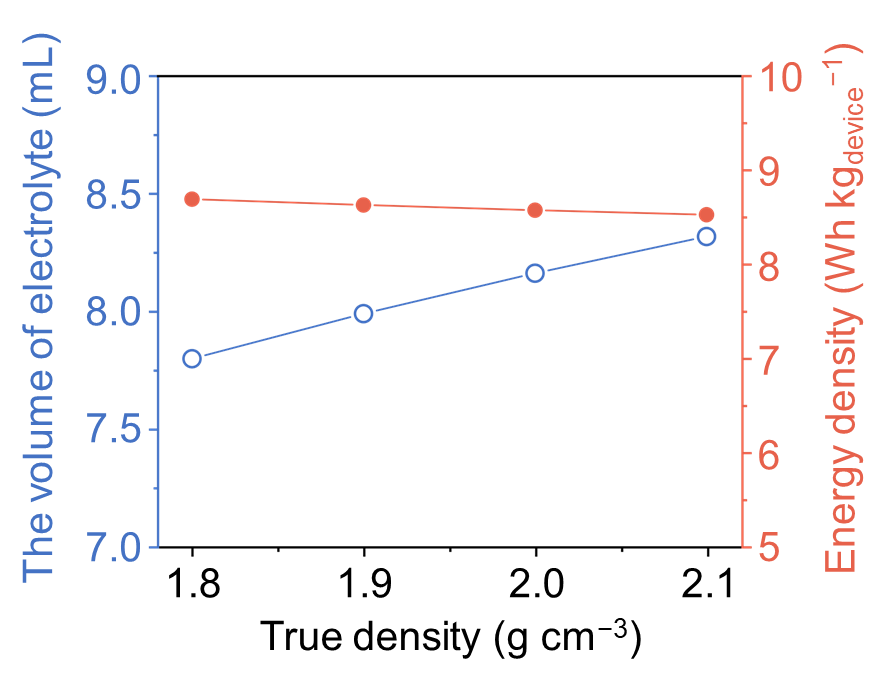


**Fig. S12** Required volume of electrolyte and *E*_device_ of supercapacitors using AC materials with different true densities

For true density of ACs in between 1.8 and 2.1 g cm^−3^, the simulated *E_device_* revealed minimal variation. Therefore, a true density of 2.0 g cm^−3^ was adopted for the 43 AC materials form literature in the subsequent analyses.


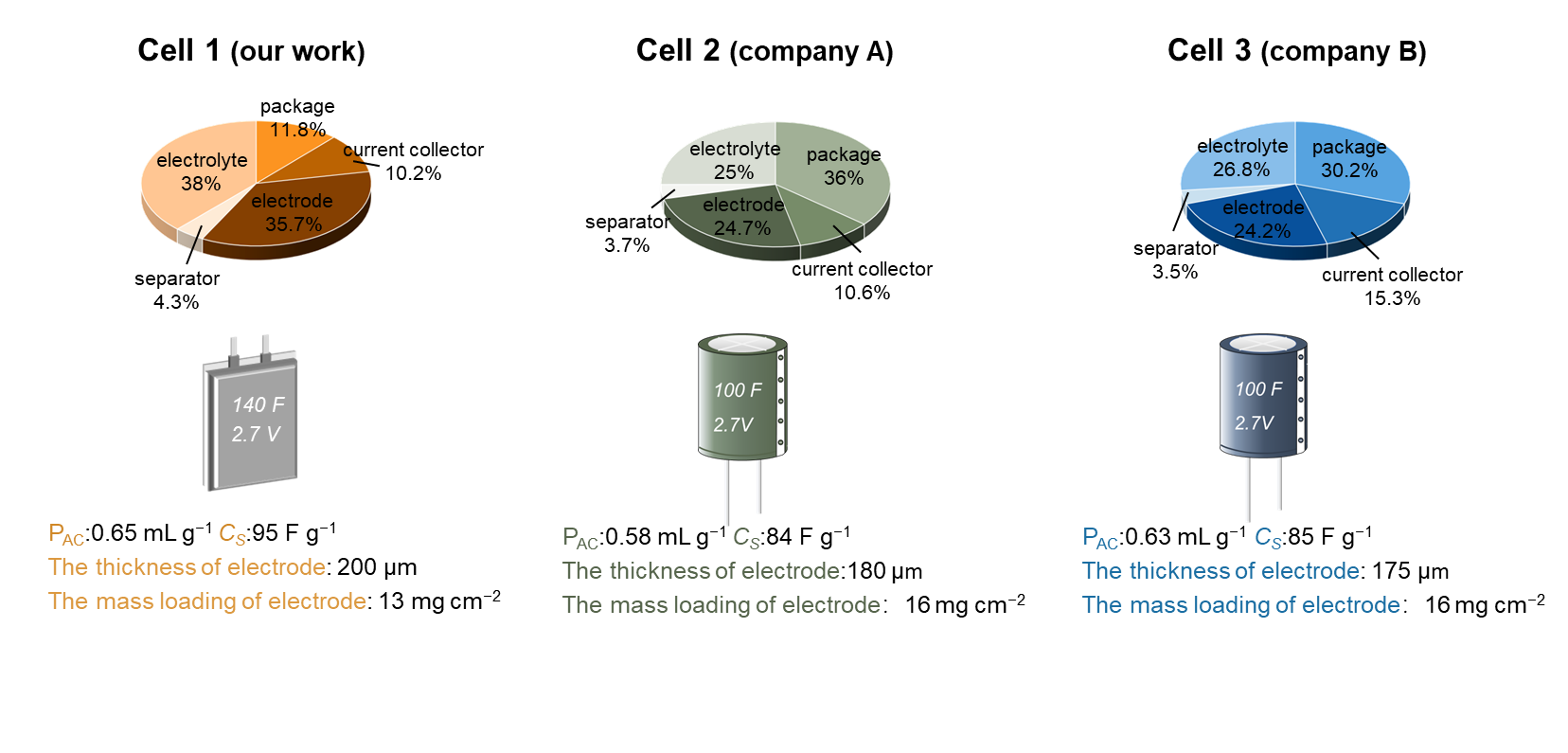


**Fig. S13** Mass distributions of the components in supercapacitor and the key parameters of AC materials and electrodes. Commercial supercapacitors were disassembled to analyze their mass distribution and properties of AC materials


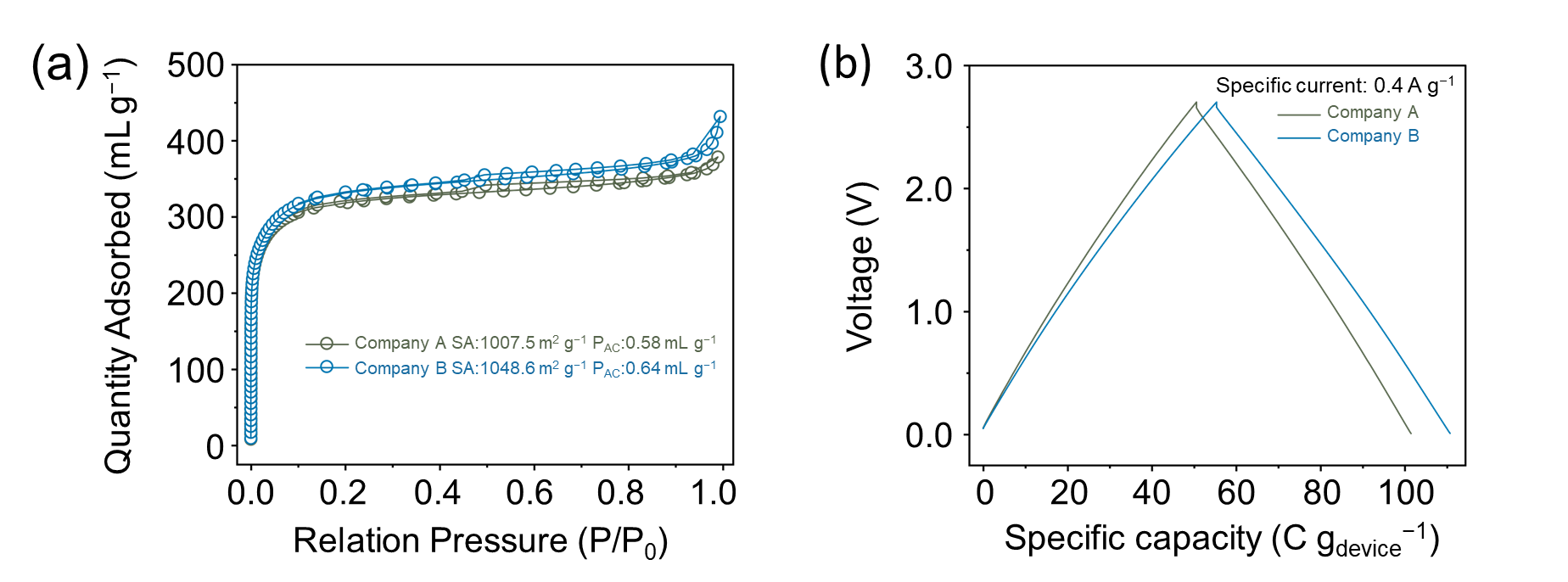


**Fig. S14** **a** N_2_ adsorption‒desorption isotherms of the AC materials of different cylindrical supercapacitors. **b** Charging and discharging curves of cylindrical supercapacitors


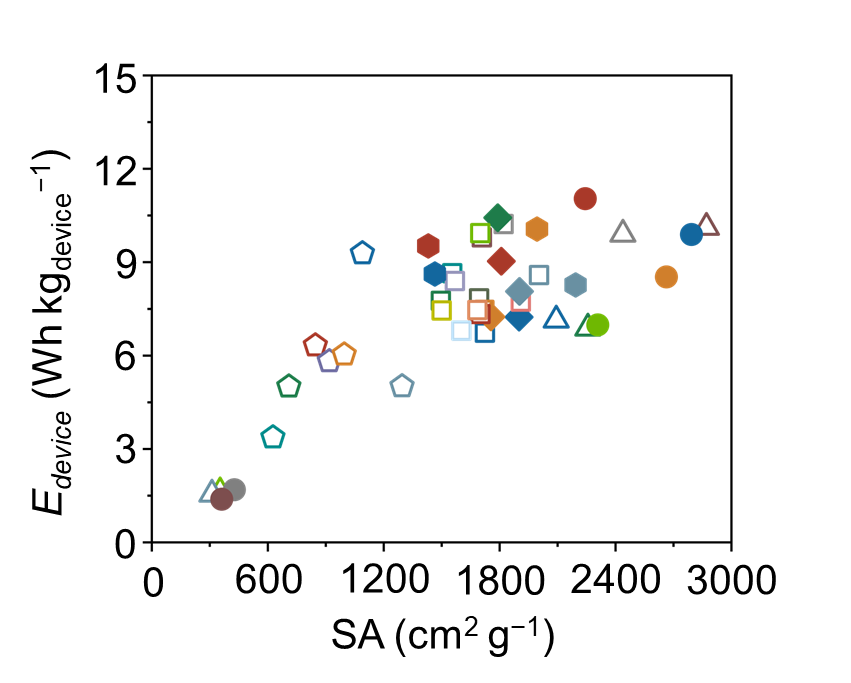


**Fig. S15** The plot of *E*_devicce_ *vs.* specific surface area of AC materials


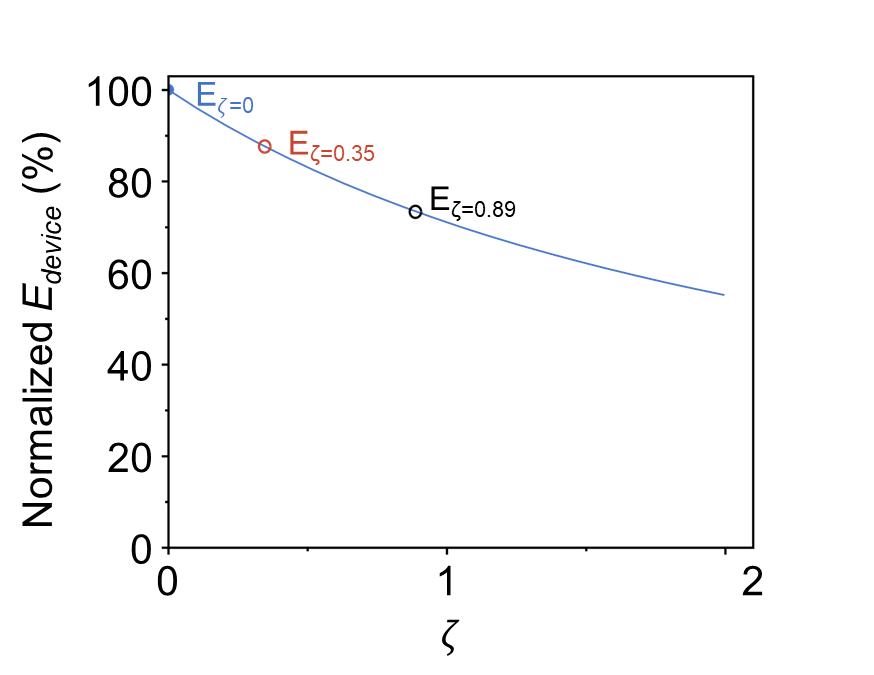


**Fig. S16** The influence of correction coefficient ($\zeta$) on *E_device_*

Using *C_S_* = 95 F g^−1^, P_AC_ = 0.65 mL g^−1^, and *m_a_* = 13 mg cm^−2^, the influence of the packaging factor $\zeta$ on the *E*_device_ is investigated. For a ~140 F supercapacitor cells, the $\zeta$ of pouch package and cylindrical package is 0.35 and 0.89, respectively, corresponding to 87% and 73% of normalized *E_device_* (when $\zeta$ = 0). Usually, when larger supercapacitor cell is assembled, the influence of $\zeta$ would be decrease.


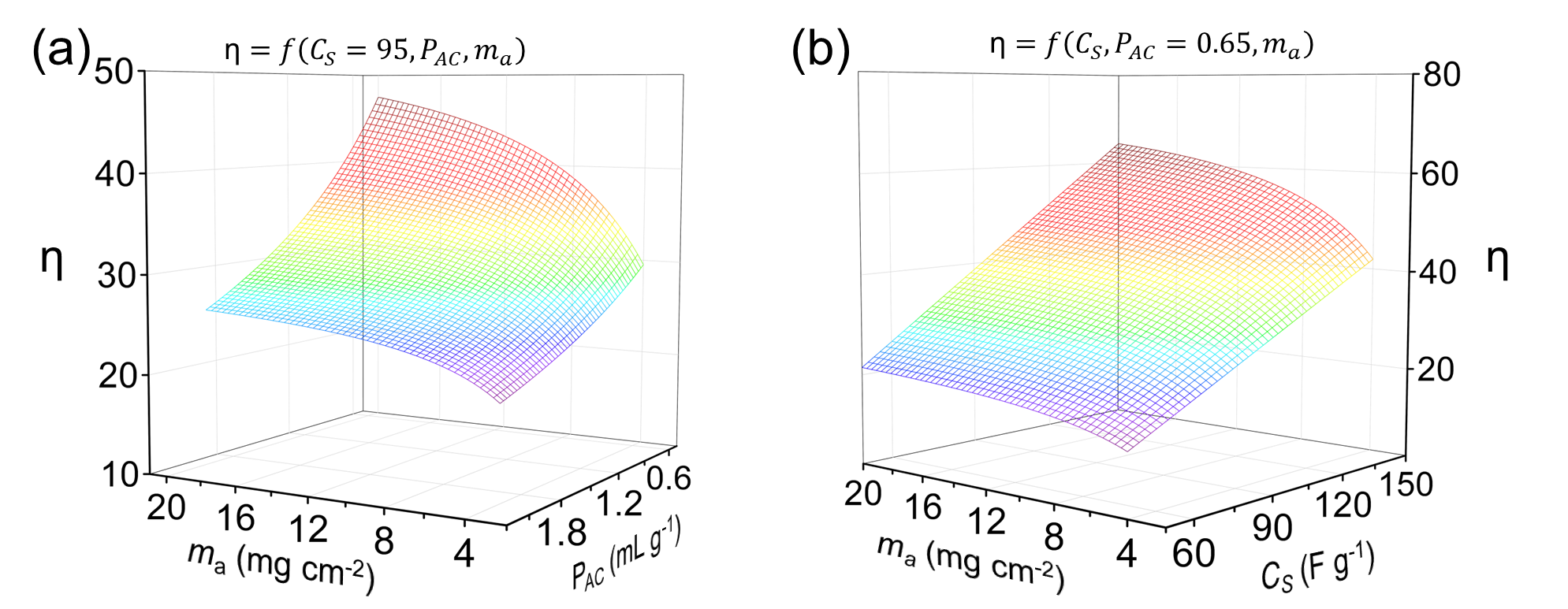


**Fig.** **S17** **a** The 3D function relation plots of (a) *m_a_* (mg cm^−2^) and P_AC_ (mL g^−1^) with respect to η, where *C_S_* = 95 F g^−1^. **b** *m_a_* (mg cm^−2^) and *C_S_* (mL g^−1^) to η, fixing the P_AC_ = 0.65 mL g^−1^

**
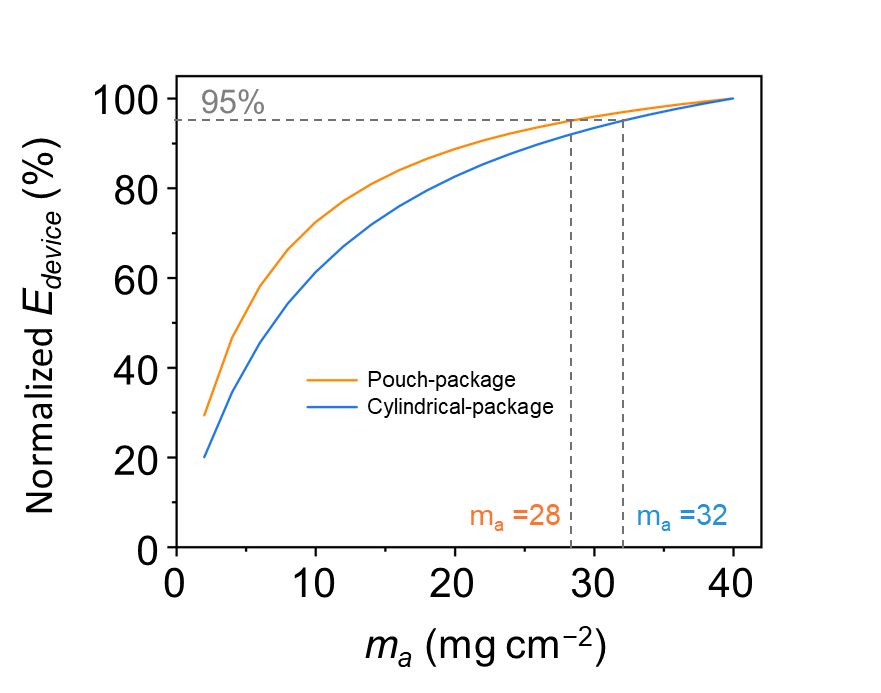
**

**Fig. S18** The *E_device_* of AC (*C_S_* = 95 F g^−1^, P_AC_ = 0.65 mL g^−1^) across varying mass loadings (ranging from 2 to 40 mg cm⁻², double-side coating)

**Table S1** The parameter specification of separators

| NKK TF4030 cellulose separators | |
| --- | --- |
| thickness | 30$\text{μm}$ |
| width | 60$\text{ mm}$ |
| density | 0.421 g cm^−3^ |
| porosity | 73$\%$ |
| strength | $\geq$7.8 $\text{N/}$15$\text{mm}$ |
| pH | 5.5-8.0 |

**Table S2** Characterization of the AC electrode

| The parameters of commercial activated carbon electrode | |
| --- | --- |
| mass loading (mg cm^−2^) | 6.5 |
| mass ratio of electrode  (AC: conductive carbon: binder) | 95:3:2 |
| thickness of the electrode ($\text{μm}$) | 100 |
| true density (g cm^−3^) | 1.94 |
| accumulative pore volume (mL g^−1^) | 0.65 |
| specific surface area (m^2^ g^−1^) | 1051 |
| the volume ratio of stacked pores to the electrode | 26.7% |
| V_pore_ (mL) | 8.15 |
| V_AC_ (mL) | 4.04 |
| V_P_ (mL) | 2.67 |
| Vs (mL) | 1.36 |

**Table S3** The densities and ionic conductivities of electrolytes

| Concentrations | Density (g mL^−1^) | Ionic conductivity (mS cm^−1^) |
| --- | --- | --- |
| 0.25 M Net_4_BF_4_ in ACN | 0.807 | 24.07 |
| 0.5 M Net_4_BF_4_ in ACN | 0.825 | 36.13 |
| 1.0 M Net_4_BF_4_ in ACN | 0.864 | 52.9 |
| 1.5 M Net_4_BF_4_ in ACN | 0.898 | 58 |

**Table S4** The accumulative pore volume, specific capacitance, BET surface area, electrolyte and predicted *E_device_* for 43 AC materials from literature

| Carbon Sample | Accumulative pore volume (mL g^−1^) | Specific capacitance (F g^−1^) | BET surface area  (m^2^ g^−1^) | Electrolyte | *^#^E*_device_  (Wh $\text{kg}_{\text{device}}^{\mathbf{-}\text{1}}$) | Refs. |
| --- | --- | --- | --- | --- | --- | --- |
| YP-50F  YP-80F  PW-400  SC-1800  ACS-PC  EL-104  EL-106  ACC-10  ACC-15  ACC-20  APC-700-1  APC-700-2  APC-800  APC-950  APC-1000  APC-1050  APC-1100  AEL-1000  AEL-1100  AEL-1200 | 0.70  1.10  0.80  0.80  0.90  0.80  0.90  0.40  0.50  0.80  0.80  0.80  0.90  0.80  0.80  0.80  0.90  0.80  0.70  0.70 | 94.3  95.7  84.3  128  135.3  93.5  93.7  98  105.1  107.5  122.6  124.3  117.1  108.2  105.1  97.1  93.8  91.8  89.8  81.8 | 1694  2264  1724  1821  1796  1721  1907  1094  1436  2004  1708  1700  1815  1555  1569  1496  1761  1701  1685  1602 | 1 M Net_4_BF_4_ in ACN | 7.84  6.85  6.73  10.22  10.40  7.47  7.21  9.26  9.50  8.59  9.79  9.93  9.01  8.64  8.40  7.76  7.21  7.33  7.46  6.80 | [S2] |
| PICA A  PICA B  PICA C | 1.63  1.12  0.71 | 115  100  90 | 2315  2100  1500 | 1.7 M N(C_2_H_5_)_4_CH_3_SO_3_ in ACN | 6.95  7.11  7.45 | [S3] |
| CDC-700 | 1.4 | 170 | 2250 | 1 M Net_4_BF_4_ in ACN | 11.02 | [S4] |
| ND 1300-AR  ND 1300-VAC  ND 1700-AR  ND 1700-VAC | 1.11  1.11  1.23  1.30 | 22  21  24  20 | 356  314  430  364 | 1 M  Net_4_BF_4_  in ACN | 1.57  1.50  1.64  1.34 | [S5] |
| Hurd-a-1  Hurd-a-3  Hurd-a-5  Hurd-b-5  Bast-a-5  Bast-b-5 | 0.73  1.16  1.71  1.06  1.76  0.86 | 94  144  167  136  146  103 | 1910  2879  2801  2446  2671  1909 | 1.8 M Net_4_BF_4_ in PC | 7.73  10.09  9.87  9.87  8.50  8.03 | [S6] |
| CPr48  CS48  CP48  CPr15  CS15  CP15  CPr15T  CSU  CPrU | 0.25  0.58  0.29  0.21  0.45  0.25  0.2  0.57  0.28 | 62  115  50  48  93  57  32  94  60 | 850  2000  1300  713  1470  923  630  2200  1000 | 1 M Net_4_BF_4_ in ACN | 6.29  10.04  4.98  4.97  8.59  5.78  3.33  8.24  6.00 | [S7] |

**Note:** ***^#^***Energy density is simulated via the provided *E*-tool.

**S5 *E*-tool for supercapacitor**

| **Input information of materials** | | | |
| --- | --- | --- | --- |
| Electrode properties | | Sepatator | |
| active material | activated carbon (AC) | NKK TF4030 | |
| accumulative pore volume of activated carbon (mL/g) | 0.65 | porosity (%) | 73% |
| true density (g/cm^3^) | 2 | thickness (μm) | 30 |
| porosity of stacking pores | 26% | width (cm) | 6 |
| double-side mass loading (*m_a_*, mg/cm^2^) | 13 | density (g/cm^3^) | 0.421 |
| content of active material (wt.%) | 95% | Electrolyte | |
| specific capacitance (F/g) | 95 | 1 M Net_4_BF_4_ in ACN | |
| width (cm) | 4.3 | density (g/mL) | 0.8639 |
| length (cm) | 5.6 | Cuerrent collector | |
| area of single electrode (cm^2^) | 24.08 | areal density of 12 μm Al foil (mg/cm^2^) | 3.24 |
| positive pieces | 10 | Operating voltage of supercapacitor | |
| negative pieces | 11 | voltage window (V) | 2.7 |
|  | | | |
| **Supercapacitor device** | | | |
| mass of AC (m_AC_, g) | 6.57 | mass of electrolyte (V_total_) (g) | 6.91 |
| volume of AC (cm^3^) | 3.29 | mass of separator (g) | 0.78 |
| V_AC_ (cm^3^) | 4.06 | mass of current collector (Al foil) (g) | 1.64 |
| total volume of electrode (cm^3^) | 9.93 | mass of package (g) | 2.17 |
| V_P_ (cm^3^) | 2.58 | mass of binder and conductive carbon (g) | 0.35 |
| V_pore_ (cm^3^) | 6.64 | total mass of device (*M_total_*, g) | 18.42 |
| Vs (cm^3^) | 1.36 | rated capacitance (F) | 141 |
|  | | | |
| **Output results of supercapacitor cell** | | | |
| *E*_AC_ (Wh/kg) | 22.90 | conversion factor (*f*) | 0.34 |
| *E*_device_ (Wh/kg) | 7.77 |  |  |
|  | | | |
| NOTE: ^1^V_AC_ is the volume of pore in AC; V_P_ is the volume of stacking pore; Vs is the volume of pore in separator; | | | |
| ^2^The cacluating equation of energy density : *E_AC_*=1/2*C*V^2^/m_AC_; *E_device_*=1/2*C*V^2^/M_total_ | | | |

**Supplementary References**

1. S. Wang, L. Yu, S. Wang, L. Zhang, L. Chen et al., Strong, tough, ionic conductive, and freezing-tolerant all-natural hydrogel enabled by cellulose-bentonite coordination interactions. Nat. Commun. **13**(1), 3408 (2022). <https://doi.org/10.1038/s41467-022-30224-8>
2. X. Liu, D. Lyu, C. Merlet, M.J.A. Leesmith, X. Hua et al., Structural disorder determines capacitance in nanoporous carbons. Science **384**(6693), 321-325 (2024). https://doi.org/ 10.1126/science.adn6242
3. J. Gamby, P.L. Taberna, P. Simon, J.F. Fauvarque, M. Chesneau, Studies and characterisations of various activated carbons used for carbon/carbon supercapacitors. J. Power Sources **101**(1), 109-116 (2001). https://doi.org/10.1016/S0378-7753(01)00707-8
4. Y. Korenblit, M. Rose, E. Kockrick, L. Borchardt, A. Kvit et al., High-rate electrochemical capacitors based on ordered mesoporous silicon carbide-derived carbon. ACS Nano **4**(3), 1337-1344 (2010). https://doi.org/10.1021/nn901825y
5. M. Zeiger, N. Jäckel, D. Weingarth, V. Presser, Vacuum or flowing argon: What is the best synthesis atmosphere for nanodiamond-derived carbon onions for supercapacitor electrodes? Carbon. **94**(507-517 (2015). https://doi.org /10.1016/j.carbon.2015.07.028
6. W. Sun, S.M. Lipka, C. Swartz, D. Williams, F. Yang, Hemp-derived activated carbons for supercapacitors. Carbon **103**, 181-192 (2016). https://doi.org/ 10.1016/j.carbon.2016.02.090
7. C. Vix-Guterl, E. Frackowiak, K. Jurewicz, M. Friebe, J. Parmentier et al., Electrochemical energy storage in ordered porous carbon materials. Carbon **43**(6), 1293-1302 (2005). https://doi.org/ 10.1016/j.carbon.2004.12.028
